# The ESCRT regulator Did2 maintains the balance between long-distance endosomal transport and endocytic trafficking

**DOI:** 10.1371/journal.pgen.1006734

**Published:** 2017-04-19

**Authors:** Carl Haag, Thomas Pohlmann, Michael Feldbrügge

**Affiliations:** Heinrich Heine University Düsseldorf, Institute for Microbiology, Cluster of Excellence on Plant Sciences, Düsseldorf, Germany; Oregon State University, UNITED STATES

## Abstract

In highly polarised cells, like fungal hyphae, early endosomes function in both endocytosis as well as long-distance transport of various cargo including mRNA and protein complexes. However, knowledge on the crosstalk between these seemingly different trafficking processes is scarce. Here, we demonstrate that the ESCRT regulator Did2 coordinates endosomal transport in fungal hyphae of *Ustilago maydis*. Loss of Did2 results in defective vacuolar targeting, less processive long-distance transport and abnormal shuttling of early endosomes. Importantly, the late endosomal protein Rab7 and vacuolar protease Prc1 exhibit increased shuttling on these aberrant endosomes suggesting defects in endosomal maturation and identity. Consistently, molecular motors fail to attach efficiently explaining the disturbed processive movement. Furthermore, the endosomal mRNP linker protein Upa1 is hardly present on endosomes resulting in defects in long-distance mRNA transport. In conclusion, the ESCRT regulator Did2 coordinates precise maturation of endosomes and thus provides the correct membrane identity for efficient endosomal long-distance transport.

## Author summary

Eukaryotic cells rely on constant membrane trafficking to organise their intracellular compartments. Endosomes are important transport units that serve as sorting platforms during endocytosis. Internalised cargo destined for degradation is transferred via maturing endosomes towards the lysosome/vacuole. Essential for this maturation and membrane remodelling is the so-called ESCRT machinery. In highly polarised cells, like fungal hyphae, endosomes carry out an additional role: they transport various cargos such as mRNAs and protein complexes over long distances. At present it is unclear how these two seemingly independent functions are coordinated. Here we demonstrate in the plant pathogen *Ustilago maydis* that the ESCRT regulator Did2 is required for the dual function of endosomes. Loss of Did2 resulted in defective targeting of vacuolar proteins, disturbed endosomal mRNA-transport as well as altered shuttling of endosomes. Importantly, we observed that in absence of Did2 proteins typically present on static late endosomes or in vacuoles are now present on motile early endosomes. Based on these findings, we propose that Did2 specifically controls endosomal maturation and identity. Thereby, vacuolar sorting is insulated from long-distance transport. In conclusion, we provide new insights into the complex regulatory potential of the ESCRT regulator Did2 in polarised eukaryotic cells.

## Introduction

Endocytosis constitutes a fundamental eukaryotic mechanism that is required for the regulation of important cellular processes, e.g. establishment of cell polarity at the plasma membrane. Endocytosis mediates, for example, the removal of plasma membrane proteins from the cell surface for subsequent degradation in the lysosome/vacuole. More specifically, after internalisation, membrane proteins are sorted into early endosomes, which mature to late endosomes before they are targeted to the lysosomal/vacuolar compartment [1]. Important markers for the identity of the different endosomal compartments are small Rab-type GTPases. Rab5 and Rab7, for example, are specific for early and late endosomes, respectively [1].

A key component of endosomal maturation is the ESCRT complex (endosomal sorting complex required for transport), a highly evolutionary conserved multi-protein complex [2–5]. One function of this sophisticated membrane remodelling machinery is the formation of intraluminal vesicles within endosomes, resulting in the formation of multivesicular bodies. Thereby, endocytosed transmembrane proteins such as receptors are sorted for degradation in the lysosome/vacuole [3, 4].

ESCRT functions have been well-studied in *Saccharomyces cerevisiae*, where numerous components of the subcomplexes ESCRT-0 to -III were identified by defects in vacuolar protein sorting observed in the corresponding *vps* mutants [6, 7]. Vacuolar carboxypeptidases, for example, are sorted to the vacuole in an ESCRT-dependent manner. Transport of carboxypeptidase S is blocked at the vacuolar membrane in *vps* mutants and thus the enzyme does not reach the vacuolar lumen [8, 9]. ESCRT-mutants also form a so-called class E compartment, which can be stained with the lipophilic dye FM4-64. Ultrastructural analysis revealed that the class E compartment is a large aberrant accumulation of endosomal membrane stacks devoid of intraluminal vesicles [10].

An essential step for membrane remodelling is the formation of the ESCRT-III complex by multimerisation of its core components Snf7p (Vps32p, CHMP4). Its functionality strongly depends on the ATPase Vps4p that has been implicated in disassembly and recycling of the complex. In accordance, loss of Vps4p results in accumulation of ESCRT-III components on endosomal membranes [11]. Vps4p activity is modulated by a complex regulatory network containing the interaction partners Vps60p and Did2p (CHMP5 and CHMP1A, respectively) [12, 13]. This is for example achieved by direct interaction of the Vps4p MIT domain (microtubule interacting and trafficking) [14] with the C-terminal MIM motifs of Vps2p and Did2p (MIT interacting motif) [13, 14].

In the past years, it became more and more apparent that endosomes are multipurpose carriers that besides endocytosis also function in intracellular long-distance transport. This is particularly important for highly polarised cells such as neurons or fungal hyphae. In fungal hyphae endosomal cargo is comprised of entire organelles such as peroxisomes [15, 16] as well as mRNAs, ribosomes, and protein complexes [15, 17–20]. However, at present it is unclear how endocytic membrane trafficking and long-distance transport are choreographed.

**Fig 1:**
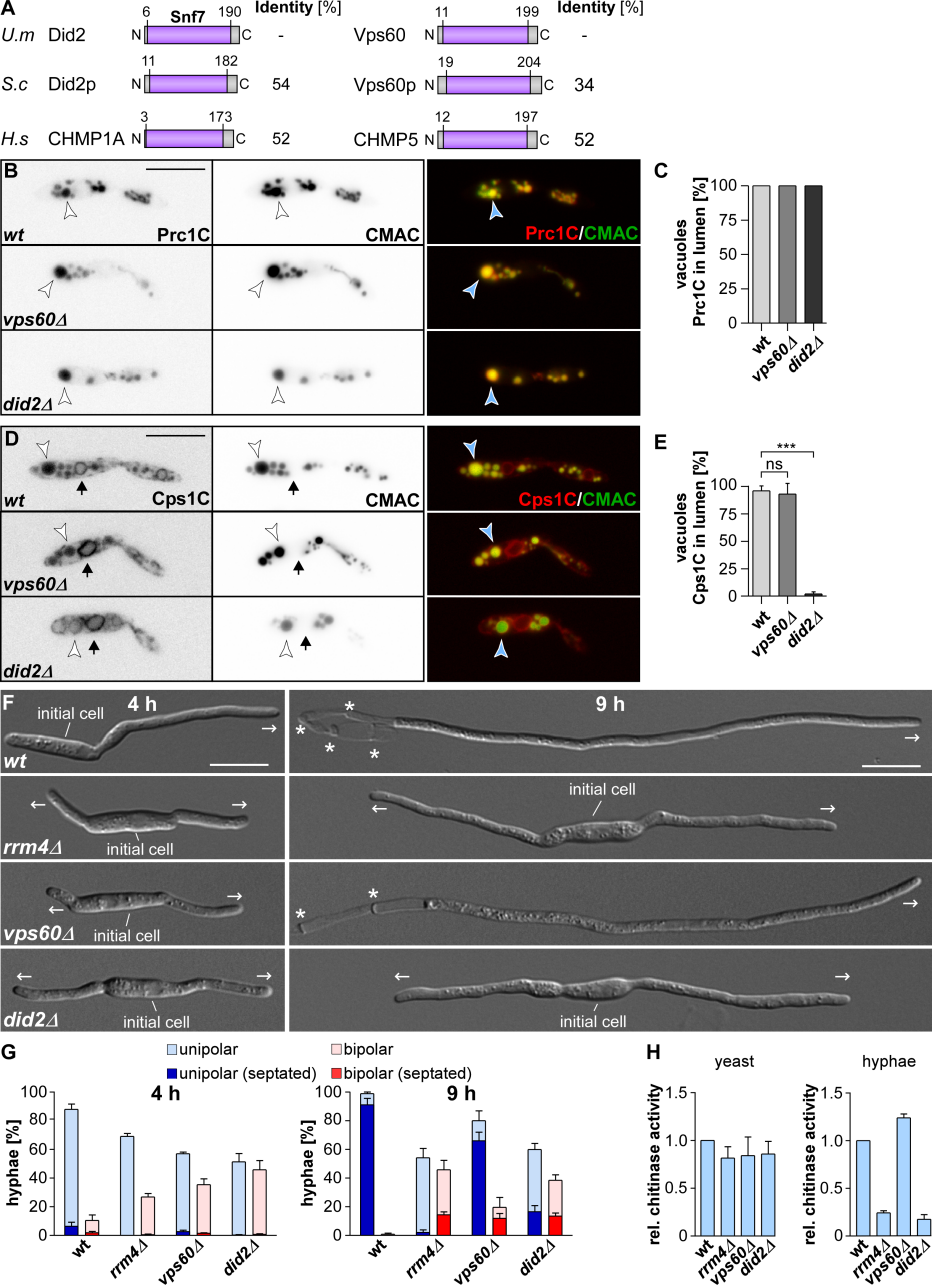
Deletion of *did2* causes defects in hyphal growth. (A) Comparison of amino acid sequence identity (>92% query coverage; BLASTp) [35] and domain architecture (SMART) [36] of ESCRT regulators Did2 and Vps60. All proteins belong to the family of Snf7 proteins (PFAM; PF03357; for accession numbers, see Materials and Methods). (B) Fluorescence micrographs of yeast cells (inverted images, left) ectopically expressing Prc1 C-terminally fused to mCherry (Prc1C, left). The amino acid sequence of *S*. *cerevisiae* Prc1p exhibits 41% identity to *U*. *maydis* Prc1 (79% query coverage). Vacuoles were stained with CMAC (centre). An overlay is shown on the right (arrowheads indicate vacuoles; size bar, 10 μm). (C) Bar chart depicting co-localization of lumenal Prc1C with CMAC-stained vacuoles (d≥1 μm). (n = 3 independent experiments; at least 39 vacuoles/strain per experiment were analysed). (D) Fluorescence micrographs of yeast cells (inverted image) ectopically expressing Cps1 C-terminally fused to mCherry (Cps1C). Cps1p from *S*. *cerevisiae* exhibits 39% identity to Cps1 from *U*. *maydis* (87% query coverage). Vacuoles were stained with CMAC (centre). An overlay is shown on the right (arrowheads indicate vacuoles, arrows mark nuclear-associated ER; size bar, 10 μm). (E) Bar chart depicting co-localization of lumenal Cps1C with CMAC-stained vacuoles (d≥1 μm; error bars, s.d.; n = 3 independent experiments, at least 44 vacuoles/strain per experiment were analysed; ***, p<0.001; ns, not significant; unpaired two-tailed t-test). (F) Growth of AB33 derivates four and nine hours post induction of filamentous growth (4 and 9 h.p.i., basal septae and growth direction are marked by asterisks and arrows, respectively; size bar, 10 μm). (G) Percentage of hyphae (4 and 9 h.p.i.): quantification of unipolarity, bipolarity and septum formation (error bars, s.e.m.; n = 3 independent experiments, > 100 hyphae/strain were counted per experiment). (H) Measurement of extracellular Cts1 activity in yeast cells and hyphae ([31]; error bars, s.e.m.; n = 3 independent experiments).

In the well-studied fungal model system *U*. *maydis*, long-distance transport of early endosomes along the microtubule cytoskeleton is essential for unipolar hyphal growth. Defects in microtubule-dependent transport or loss of Rab5a result in a characteristic bipolar phenotype, i.e. hyphae establish two growth poles at opposite ends of the initial cell (Fig 1) [21–23]. Endosomes are transported along an antiparallel array of microtubules. Minus-end directed movement is mediated by cytoplasmic dynein and plus-end directed movement by the kinesin-3 type motor Kin3 [24, 25].

Recently, we discovered a close link between endosomal transport and mRNA trafficking [26]. Initially, we observed that loss of the key RNA-binding protein for microtubule-dependent mRNA transport, Rrm4, also caused defects in unipolar hyphal growth [27, 28]. Studying the mode of transport revealed that Rrm4 was hitchhiking on shuttling Rab5a-positive endosomes [24]. Endosomal association is mediated by direct interaction of Rrm4 with the endosomal FYVE domain-containing protein Upa1 [29, 30]. *In vivo* UV cross-linking of mRNAs covalently to Rrm4 identified septin *cdc3* mRNA as target of the Rrm4 transport machinery [17]. Further studies revealed that not only septin mRNA but also the encoded protein was transported on early endosomes [18]. Endosomal localisation is important for the assembly of heteromeric septin complexes and this ensures efficient formation of septin filament gradients at the growth pole of hyphae [18, 20]. Loss of Rrm4 resulted in defects in endosomal localisation of septins and consequently aberrant septin gradient formation. Thus, Rrm4-mediated mRNA transport was important for the correct subcellular localisation of the encoded proteins [18, 20].

In UV-crosslinking experiments using Rrm4 as bait we identified *did2* and *vps60* mRNAs as potential targets. Therefore, we addressed a potential molecular link between endosomal mRNA transport and the subcellular function of ESCRT components during hyphal growth.

## Results

### Did2 is needed for efficient vacuolar sorting during yeast growth

In order to identify mRNA targets of Rrm4 we previously performed *in vivo* UV cross-linking experiments and discovered a number of putative candidates [17, 31]. Among these candidates were potential orthologues of the ESCRT regulators Did2 and Vps60 from *S*. *cerevisiae* (UMAG_05607 and UMAG_05282, respectively; S1A Fig) [10–12]. Sequence comparisons revealed that both proteins share about 50% sequence identity with the animal orthologues containing a common Snf7p-like fold (Fig 1A) [32]. The regions of the MIM1 [33] as well as the MIM5 motif [34] were also conserved in *U*. *maydis* Did2 and Vps60, respectively (S1B and S1C Fig).

For functional analysis we generated deletion strains using homologous recombination in *U*. *maydis* [37]. Consistent with findings in *S*. *cerevisiae*, Vps60 and Did2 were dispensable for budding growth, but loss of both proteins resulted in reduced resistance against cell wall stress (S2A and S2B Fig) [38].

To address the role in membrane trafficking towards the vacuole we performed FM4-64 uptake assays and stained the vacuolar lumen with the fluorescent dye CMAC [12, 29]. Loss of Did2 or Vps60 did not prevent uptake and accumulation of FM4-64 in vacuolar membranes, showing that endocytosis and vacuolar trafficking were not abolished. However, *did2Δ* cells exhibited weaker staining with FM4-64 and CMAC indicating a slight defect in vacuolar trafficking (S2C Fig). Consistently, internalisation of FM4-64 was delayed in the absence of Did2 (S2D and S2E Fig).

For both strains the morphology of vacuoles was not altered and loss of neither Vps60 nor Did2 caused the formation of aberrant membrane accumulations in the vicinity of vacuoles (S2C Fig). Thus, unlike in *S*. *cerevisiae* no class E-like compartment was formed [10].

Further phenotyping revealed that *did2Δ* strains formed crinkled colonies and yeast cell length was slightly reduced (S3A and S3D Fig). Crinkled colony morphology is indicative for cell division defects, but in liquid culture *did2Δ* mutants did not exhibit a strong cell separation defect as it is known for Kinesin-3 mutants (S3A–S3C Fig) [39]. Another characteristic of ESCRT mutants in *S*. *cerevisiae* is the aberrant sorting of vacuolar proteases. Two commonly analysed markers are the vacuolar proteases Prc1p and Cps1p. In ESCRT mutants Prc1p is still sorted to the vacuole but also exported to the culture medium. In case of Cps1p, the protease does not reach the vacuolar lumen any longer but is retained in the vacuolar membrane [8, 40].

The vacuolar localisation of Prc1 was unaffected by the deletion of *vps60* and *did2* in *U*. *maydis* (Fig 1B and 1C). However, in contrast to Vps60, Did2 was required for efficient sorting of Cps1 to the lumen of the vacuole (Fig 1D and 1E). Both observations were consistent with work previously done in *S*. *cerevisiae* [8, 40]. These results verified bioinformatic predictions and are consistent with the conserved biological roles of these proteins as ESCRT components. In summary, loss of Did2 and Vps60 did not cause a strong pleiotropic phenotype in the yeast form of *U*. *maydis* but rather resulted in specific defects related to ESCRT-III functions as exemplified by the observed vacuolar protein sorting defect.

### Did2 is important for unipolar growth and unconventional secretion of Cts1

Next we studied the role of the ESCRT-III regulators during hyphal growth, a phase in which Rrm4-dependent endosomal mRNA transport is needed for efficient unipolar growth and unconventional secretion of the endochitinase Cts1 (Fig 1F–1H) [29, 31]. The latter was discovered due to the fact that loss of Rrm4 resulted in intracellular accumulation of Cts1. Most likely, transport of *cts1* mRNA is needed for Cts1 protein export. Interestingly, it was recently shown that secretion of Cts1 did not follow conventional ER/Golgi apparatus-dependent secretion [41, 42].

In the laboratory strain AB33 the key transcription factor for hyphal growth bE/bW is under control of the nitrogen regulatable promoter P_nar1_. Thus, the switch from yeast to hyphal growth can be induced by altering the nitrogen source of the medium [43]. Wildtype hyphae grow unipolar and insert septa at defined time intervals resulting in the formation of cytoplasm free sections at the basal pole (Fig 1F). Loss of Vps60 resulted in increased amounts of bipolar cells at four hours post induction (Fig 1F and 1G). At later time points however, *vps60Δ* hyphae switched to unipolar growth (Fig 1F and 1G). In contrast, deletion of *did2* caused a phenotype similar to *rrm4Δ* strains, as the increase of bipolar cells did not reverse during prolonged induction and Cts1 secretion was reduced (Fig 1F–1H) [31, 41]. Since loss of Did2 induced an overall stronger phenotype, which in some aspects closely resembled loss of Rrm4, we focused on the ESCRT-III regulator Did2. In essence, Did2 is essential for undisturbed unipolar growth of hyphae suggesting a functional role in microtubule-dependent transport. This enabled us to study the role of ESCRT-III regulator in highly polarised cells that depend on long-distance transport.

### Endosomal shuttling of Did2 is independent of Rrm4

As an ESCRT component, Did2 is localised on endosomes in different fungal organisms [10, 44]. Furthermore, Rrm4-mediated transport of mRNAs was crucial for the subcellular localisation of the encoded proteins [18]. Hence, given that the *did2* mRNA was a potential target of Rrm4 we also addressed whether the subcellular localisation of Did2 protein was Rrm4-dependent. Since the N-terminus of Did2 is required for endosomal localisation, we generated strains expressing a C-terminal fusion of Did2 with Gfp under control of the endogenous promoter (Did2G; enhanced version of Gfp, Clontech) [10, 32]. Although in other fungi the C-terminal fusion was not functional this did not interfere with the subcellular localisation of the protein [10, 44]. Consistently, strains expressing Did2G did not rescue the mutant phenotypes of *did2Δ* strains (S4A–S4D Fig) but could still be used for localisation studies.

**Fig 2:**
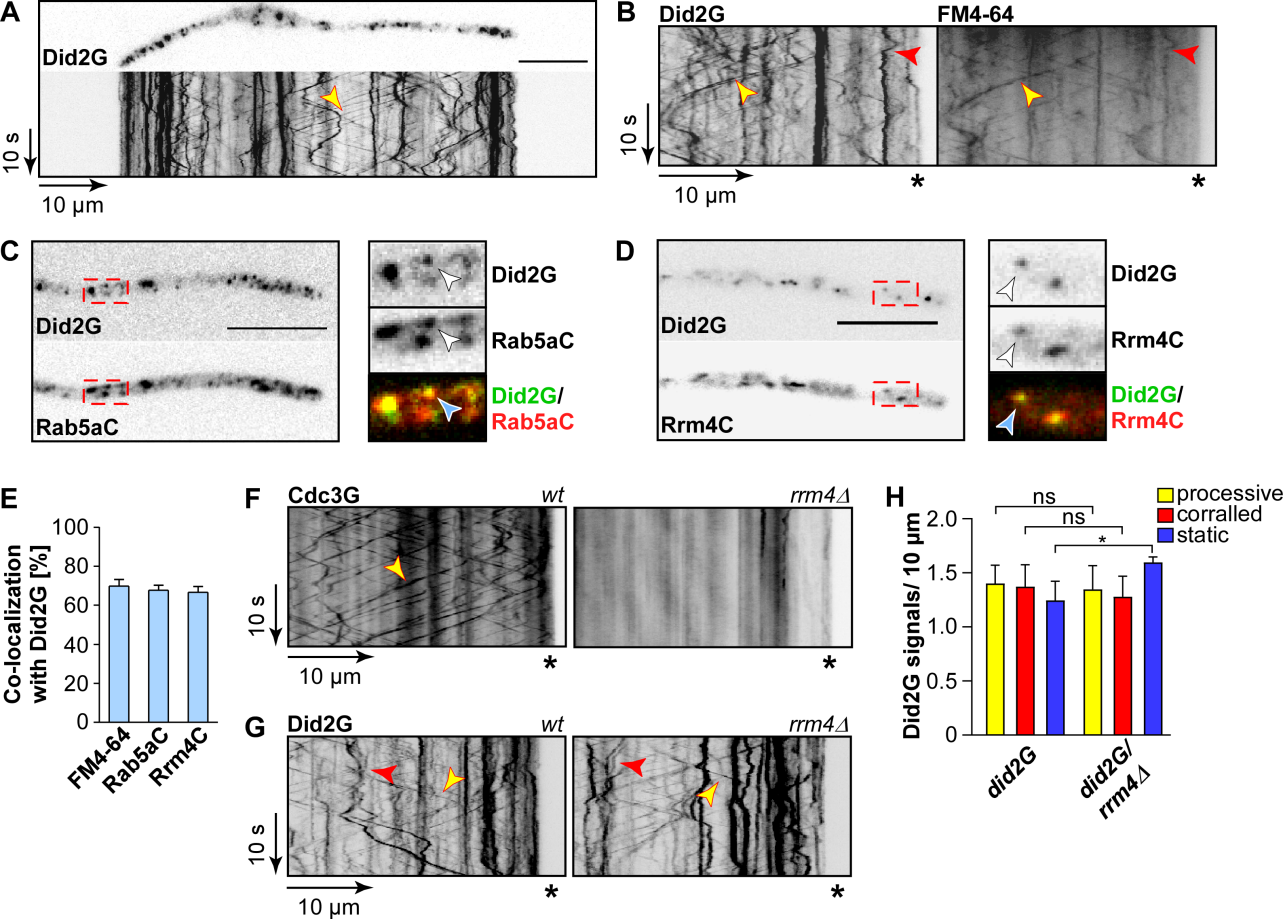
Endosomal localisation of Did2 is independent of Rrm4. (A) Fluorescent micrograph and corresponding kymograph of a hypha (6 h.p.i.) expressing Did2G. Bidirectional movement of signals is shown as diagonal lines (yellow arrowhead; size bar, 10 μm; S1 Video). (B) Dynamic co-localisation study of Did2G (left) with FM4-64 (right). Fluorescence signals were detected simultaneously using dual colour imaging. Processive co-localising signals are marked by yellow arrowheads, corralled signals by red arrowheads. Position of hyphal tips are marked by asterisks. (C-D) Co-localisation studies of Did2G with Rab5aC (C) and Rrm4C (D). Fluorescence micrographs were acquired by dual color imaging. The boxed areas are shown enlarged with the Gfp- and mCherry-signal, as well as an overlay of both images. Co-localising signals are marked by arrowheads (for corresponding kymographs, see S5A and S5B Fig). (E) Percentage of signals of the respective endosomal marker exhibiting co-localisation with Did2G (error bars, s.e.m.; n = 3 independent experiments; 6 hyphae/ strain were analysed). (F) Kymographs of hyphae expressing Cdc3G in the *rrm4* wildtype background (left) and *rrm4Δ* (right). (G) Kymographs of hyphae expressing Did2G in the *rrm4* wildtype background (left) and *rrm4Δ* (right); processive signals are marked by yellow arrowheads, corralled signals by red arrowheads and static signals by blue arrowheads; hyphal tips are marked by asterisks). (H) Bar chart depicting amount of Did2G signals per 10 μm hyphae (error bars, s.e.m; n = 3 independent experiments; 10 hyphae/strain were analysed per experiment); *, p<0.05; ns, not significant (unpaired two-tailed t-test).

In hyphae, Did2G particles shuttled bidirectionally throughout the whole hypha and accumulated in distinct static spots (Fig 2A; S1 Video). Dual colour imaging revealed that most shuttling units co-localise with endosomal markers, such as the lipophilic dye FM4-64 and Rab5aC (N-terminal fusion to mCherry; ectopical expression at the i*p*^*S*^ locus). This indicated that the moving Did2G particles were indeed early endosomes (Fig 2B and 2C; S5A Fig). Consistently, Did2G largely co-localised with Rrm4-mCherry (fused C-terminally to mCherry), proving that both proteins shuttle on identical endosomes in hyphae (Fig 2D and 2E; S5B Fig) [18, 45]. The static structures labelled by Did2G were also labelled by FM4-64, indicating that these structures are most likely late endosomes or vacuoles (Fig 2B; see S2D and S2E Fig for comparison).

As shown previously, the subcellular localisation of Cdc3G (fused N-terminally to eGfp) [18] on shuttling endosomes was dependent on Rrm4 (Fig 2F). However, deletion of *rrm4* did not alter the bidirectional movement of Did2G indicating that endosomal mRNA transport is not needed for the endosomal localisation of this ESCRT regulator (Fig 2G and 2H). Thus, unexpectedly, the Rrm4 transport machinery is not directly involved in determining the endosomal localisation of the ESCRT regulator Did2.

### Endosomal mRNA transport is disturbed in the absence of Did2

**Fig 3:**
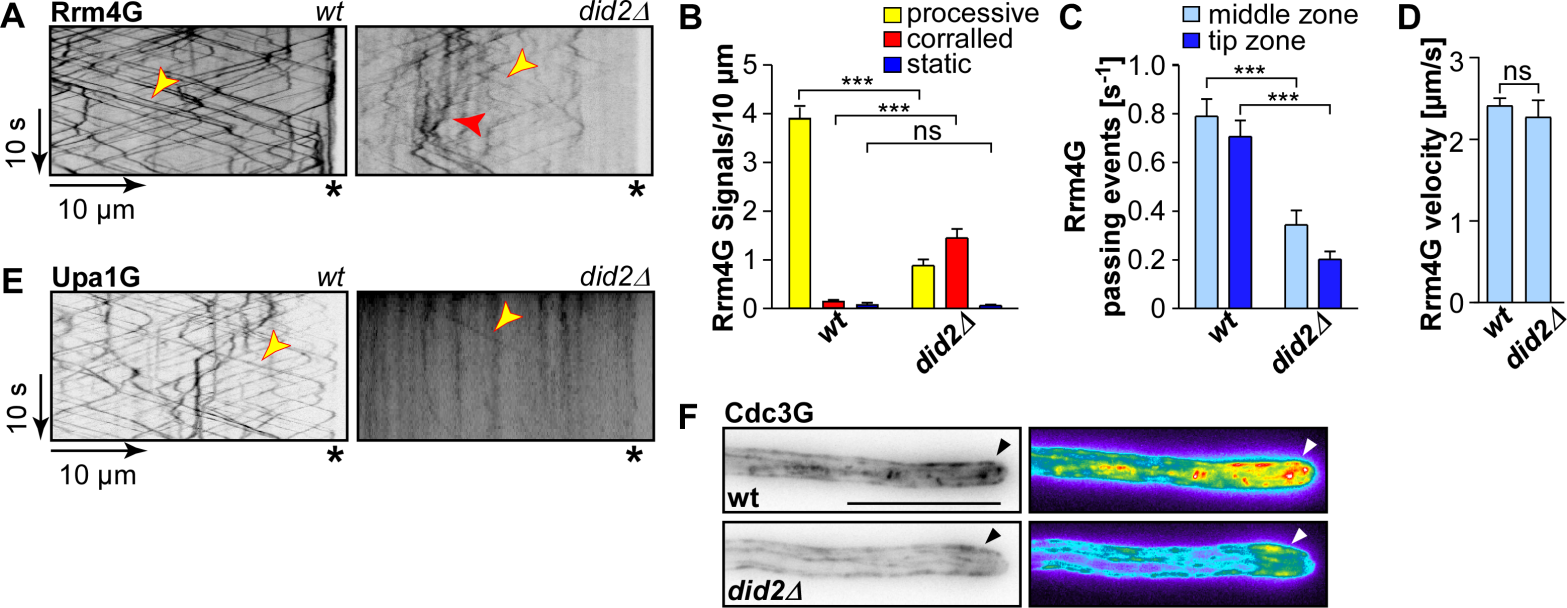
Rrm4-mediated endosomal mRNA transport is disturbed upon deletion of *did2*. (A) Kymographs of hyphae (6 h.p.i) expressing Rrm4G in the *did2* wildtype background (left) and *did2Δ* (right); processive signals are marked by yellow arrowheads and corralled signals by red arrowhead; hyphal tips are marked by asterisks; S2 Video). (B) Bar chart depicting the amount of Rrm4G signals per 10 μm hyphae (error bars, s.e.m.; N = 15 for wildtype hyphae and 16 for *did2Δ* hyphae, derived from four independent experiments were analysed, signals were scored as processive if run length > 5 μm; ***, p<0,001; unpaired two-tailed t-test). (C) Number of passing processive Rrm4G signals (run length > 5μm) over time at distinct regions within hyphae (error bar, s.e.m.; N = 15 for wildtype hyphae and 16 for *did2Δ* hyphae, derived from four independent experiments were analysed; ***, p<0.001; unpaired two-tailed t-test). (D) Bar chart depicting the velocity of processive Rrm4G signals (error bar, s.d.; see (B) for number of hyphae analysed, n = 4 independent experiments, at least three hyphae/strain, corresponding to at least 24 signals, were analysed per experiment; ns, not significant; unpaired two-tailed t-test). (E) Kymographs of hyphae (6 h.p.i) expressing Upa1G (*did2* wildtype allele left and *did2Δ* right; processive signals are marked by yellow arrowheads; hyphal tips are marked by asterisks). (F) Hyphal tip of strains expressing Cdc3G (wildtype and *did2Δ* allele are compared at the top and bottom, respectively, tip gradients are marked by arrow heads; size bar 5 μm). False colour image of maximum projection of acquired z stacks (black/blue, low to red/white high intensities).

Unipolar growth of hyphae depends on endosomal transport, particularly mRNA transport. To test an alternative explanation for the observation that Did2 is important for unipolar growth, we investigated whether endosomal transport of Rrm4 was affected in the absence of Did2. Studying Rrm4 fused to Gfp [46] in *did2Δ* strains showed that transport of Rrm4 was indeed disturbed. Processive movement of Rrm4G was reduced while the events of corralled movement increased significantly (Fig 3A–3C; S2 Video). The velocity of processive Rrm4 signals did not change in the absence of *did2* suggesting that residual processive movement still showed a connection to endosomes (Fig 3D). These observations showed that Did2 might be crucial for Rrm4-dependent endosomal mRNA transport.

Interestingly, the altered movement of Rrm4 was reminiscent of the loss of Upa1, a protein that is involved in linking Rrm4 to endosomes [29]. Indeed, endosomal movement of Upa1G (functional C-terminal Gfp fusion) [29] was hardly detectable in *did2Δ* (Fig 3E). Consequently, transport of Rrm4-dependent endosomal cargo such as mRNAs and septins should also be disturbed. Microscopic analysis confirmed that endosomal transport of the mRNA markers Pab1 fused to Gfp (Pab1G) [17], and the cargo protein septin Cdc3G [18] was impaired in *did2Δ* strains (S5C and S5D Fig). In accordance with reduced Rrm4 transport, residual movement of all the marker proteins was still processive, but reduced in signal strength. Consistently, without Did2 the Cdc3G gradient formation at the hyphal tip was also affected (Fig 3F) [18, 20]. Taken together, these results can explain why *did2Δ* is disturbed in unipolar growth and secretion of Cts1: loss of Did2 causes defects in endosomal transport of mRNA most plausibly due to defects in Upa1-dependent recruitment of Rrm4 to endosomes.

### Did2 depletion reduces the amount of shuttling endosomes and their processive movement

**Fig 4:**
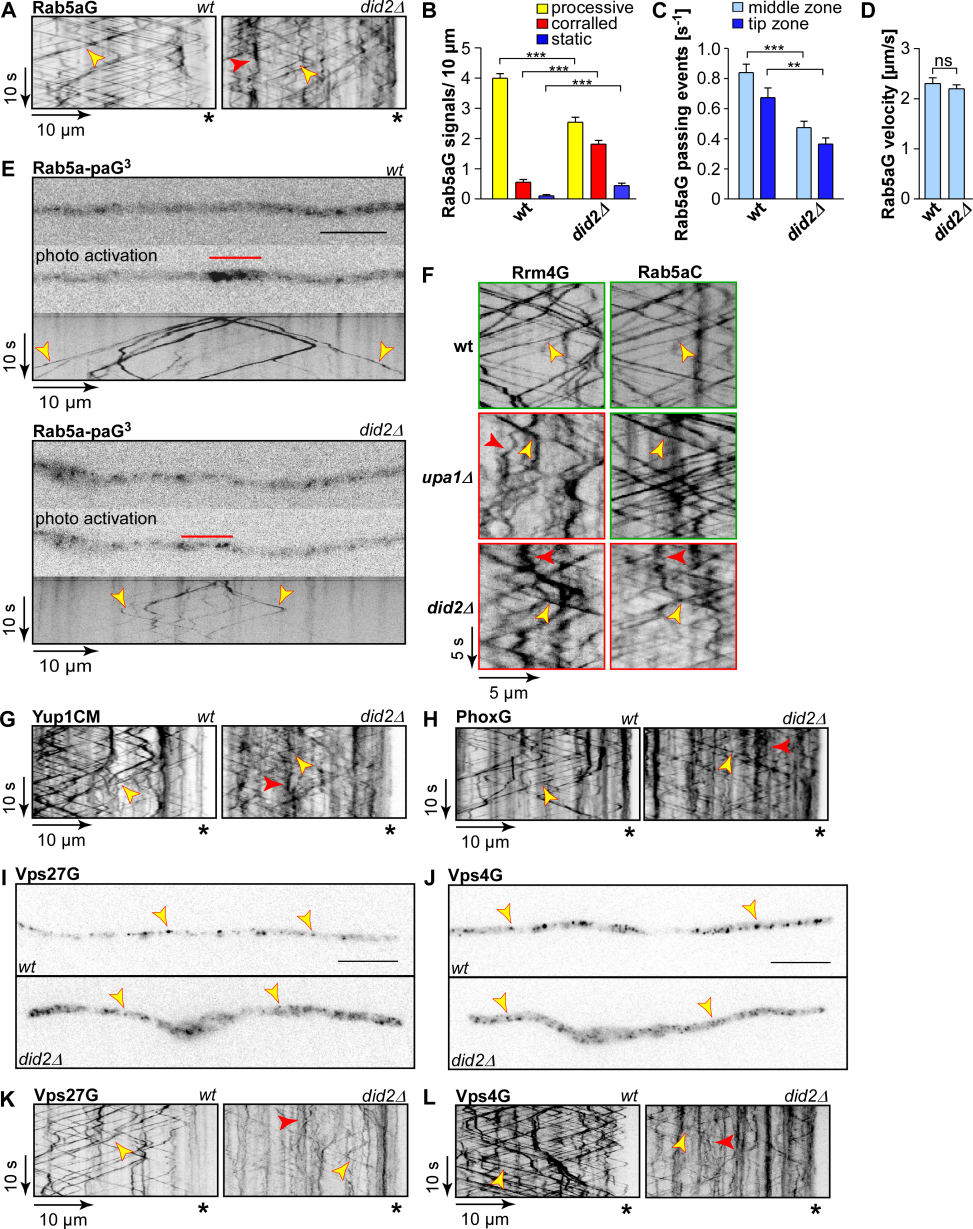
Loss of Did2 causes defects in processive endosomal movement. (A) Kymographs of hyphae (6 h.p.i) expressing Rab5aG in *did2* wildtype background (left) and *did2Δ* (right); processive signals are marked by yellow arrowheads and corralled signals by red arrowheads; hyphal tips are marked by asterisks, S3 Video). (B) Bar chart depicting the amount of Rab5aG signals per 10 μm hyphae (error bars, s.e.m..; N = 20 wildtype hyphae and 18 *did2Δ* hyphae derived from four independent experiments, signals were scored as processive if run length > 5 μm; *** p<0,001; unpaired two-tailed t-test). (C) Number of passing processive Rab5aG signals (run length > 5μm) over time at distinct regions within hyphae (error bar, s.e.m.; number of hyphae analysed given in (B);. ** p<0.01; *** p<0.001; unpaired two-tailed t-test). (D) Bar chart depicting the velocity of processive Rab5aG signals (error bar, s.d.; number of hyphae analysed given in (B), n = 4 independent experiments, at least three hyphae/strain, corresponding to at least 68 signals, were analysed per experiment; ns, not significant, unpaired two-tailed t-test). (E) Fluorescence micrographs of strain expressing photoactivatable Rab5a-paG^3^ before and after photoactivation (red line). Corresponding micrograph recorded after photoactivation is shown below (wildtype allele of *did2* top panel, *did2Δ* bottom panel; size bar 10 μm; S4 Video). (F) Dynamic co-localisation studies of Rrm4G (left) with Rab5aC (C, right). Fluorescence signals were detected simultaneously using dual colour imaging. Processive co-localizing signals and corralled signals are marked by yellow and red arrowheads, respectively. For better comparison, kymographs depicting normal movement are framed in green and those with aberrant movement in red. (G-H) Kymographs of hyphae (6 h.p.i) expressing Yup1CM (G) and PhoxG (H) in the*did2* wildtype background (left) and *did2Δ* (right); processive signals are marked by yellow arrowheads and corralled signals by red arrowhead; hyphal tips are marked by asterisks. (I-J) Fluorescence micrographs of hyphae expressing Vps27G (I) and Vps4G (J) in the *did2* wildtype background (top) and *did2Δ* (bottom). Vps27G and Vps4G-particles are marked by yellow arrowheads; size bar 10 μm. (K-L) Kymographs of hyphae (6 h.p.i) expressing Vps27G (K) and Vps4G (L) in the *did2* wildtype background (left) and *did2Δ* (right); processive signals are marked by yellow arrowheads and corralled signals by red arrowhead; hyphal tips are marked by asterisks.

To address whether Did2 is required specifically for endosomal mRNA transport we studied further endosomal marker proteins like Rab5aG (N-terminal fusion to Gfp; ectopically expressed at the i*p*^*S*^ locus) [25]. Loss of Did2 caused a decrease in processive movement and an increase in corralled movement of Rab5a as was observed for Rrm4 (Fig 4A–4C, S3 Video; Fig 3A). The velocity of the remaining processive Rab5a-positive endosomes was not disturbed (Fig 4D). In order to analyse individual signals with reduced background staining we generated strains expressing a version of Rab5a fused to three photoactivatable Gfps (Rab5a-paG3). Comparing wildtype and *did2Δ* strains clearly showed that processive movement was reduced in the absence of Did2, irrespective of the investigated region within hyphae (Fig 4E, S4 Video; S6A Fig). Finally, we compared the processive movement of Rrm4G and Rab5aC between *upa1Δ* and *did2Δ* mutants. Whereas loss of Upa1 has a specific effect on Rrm4G-dependent endosomal transport [29], loss of Did2 has a more general effect on endosomal movement affecting both endosomal proteins (Fig 4F).

To further characterise the central role of Did2 during endosomal transport, we investigated the endosomal SNARE Yup1 as an additional marker (ectopic expression of a C-terminal fusion with mCherry and myc-epitope) [39]. Comparable to Rab5a, the absence of Did2 caused aberrant movement of Yup1CM-stained endosomes (Fig 4G). To investigate if the observed transport defects are dependent on protein/lipid interactions, we employed the Phox-domain of Yup1 as an *in vivo* biosensor for endosomal lipids that functions independent of protein/protein interactions (Phox-domain from Yup1 C-terminally fused to Gfp; expressed ectopically at the *ip*^*s*^ locus) [47]. As expected, the lipid biosensor localised on endosomes positive for FM4-64 as well as vacuolar membranes (Fig 4H; S6B and S6C Fig). In *did2Δ* hyphae, corralled movement of PhoxG increased, while processive movement was reduced and the signal intensity of shuttling particles decreased (Fig 4H). The observed transport defects were therefore not just indicative of a misrecruitment of endosomal protein markers, but were indeed a consequence of a general disturbed processive transport of endosomes.

Lastly, we also analysed the dependency of the endosomal localisation of other ESCRT proteins on *did2*. We therefore expressed functional C-terminal Gfp-fusions of the ESCRT-0 component Vps27G (46% similarity, UMAG_03862) and the AAA ATPase Vps4G (72% similarity; UMAG_01669). Both fusion proteins shuttled bidirectionally throughout hyphae on FM4-64-positive endosomes, confirming that these are indeed endosomal proteins (S6D and S6E Fig). As observed for other endosomal markers, deletion of *did2* resulted in increased corralled movement of Vps27G and Vps4G, while the intensity and processivity of shuttling signals was decreased (Fig 4I and 4L). In summary, Did2 is crucial for processive long distance movement of early endosomes.

### Loss of Did2 alters the recruitment of endosomal motor proteins

The observed defects in processive movement indicated that the active motor-dependent shuttling was altered in *did2*-deficient cells. Alterations in motor recruitment, for example, could explain the observed motility defects. We therefore studied the subcellular localisation of the plus end-directed Kin3 and the minus end-directed dynein Dyn1/2 [25]. The kinesin-3 type motor Kin3 is specific for early endosomes and its processive movement is dependent on binding of the endosomal cargo [24, 25].

**Fig 5:**
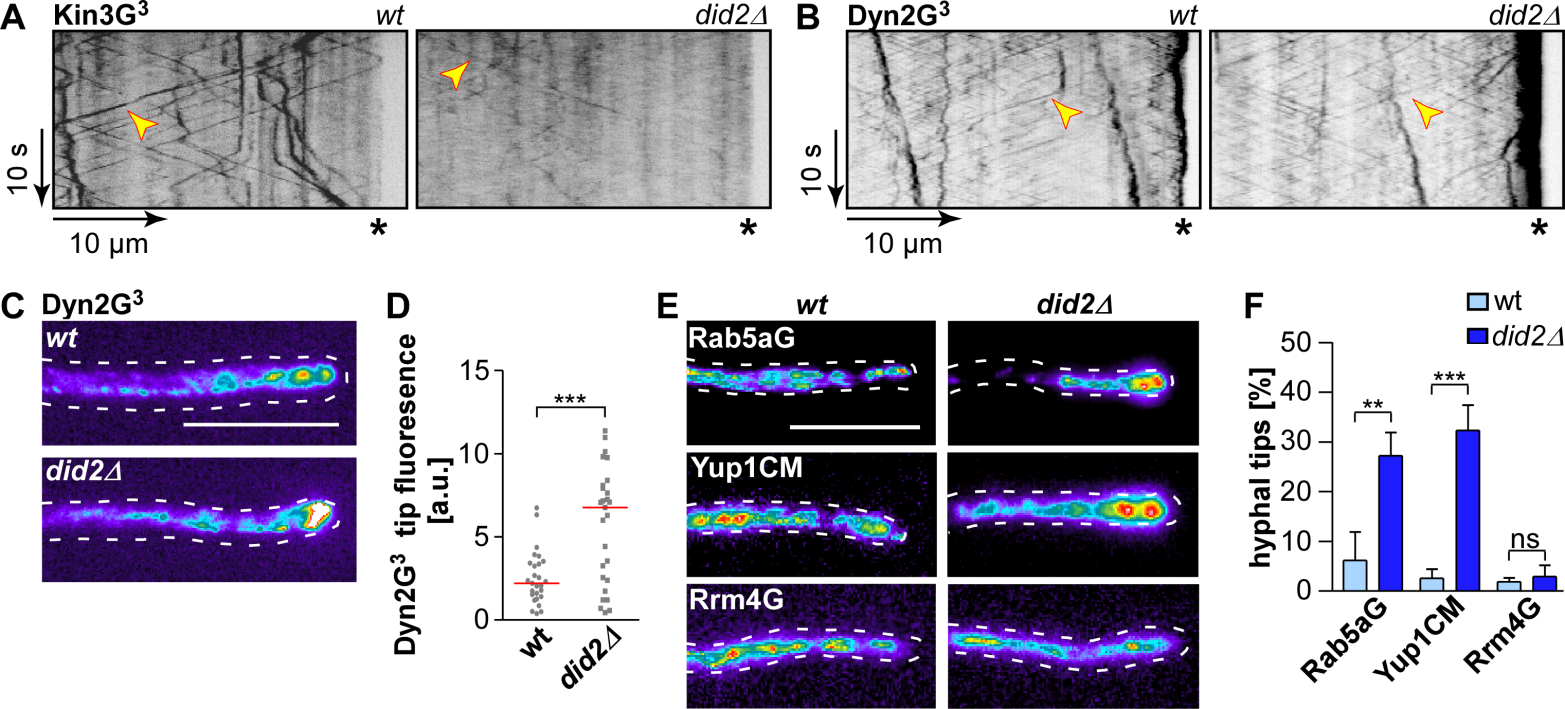
Endosomal association of the motor proteins Kin3 and Dyn2G is altered upon loss of Did2. (A) Kymographs of hyphae (6 h.p.i) expressing Kin3G^3^ in the *did2* wildtype backround (left) and *did2Δ* (right); processive signals are marked by yellow arrowheads; hyphal tips are marked by asterisks; S5 Video). (B) Kymographs of hyphae (6 h.p.i) expressing Dyn2G^3^ in the *did2* wildtype background (left) and *did2Δ* (right); processive signals are marked by yellow arrowheads; hyphal tips are marked by asterisks. (C) Fluorescence micrograph in false colours (black/blue, low to red/white high intensities) of strains expressing Dyn2G^3^ in the *did2* wildtype background (top) and *did2Δ* (bottom); size bar, 10 μm). (D) Measurement of Dyn2G^3^-fluorescence at hyphal tips. Dots represent individual measurements of Dyn2G^3^-fluorescence (total fluorescence—background) within a defined area at hyphal tips. The red line denotes the median. Tip accumulation was measured in N = 27 hyphae per strain derived from three independent experiments (arbitrary units are given; *** p>0.001; unpaired two-tailed t-test). (E) Maximum projection of z stacks in false colours (black/blue, low to red/white high intensities) of strains expressing Rab5aG (top), Yup1CM (centre) and Rrm4G (bottom) in the*did2* wildtype background (left) and *did2Δ* (right; size bar, 10 μm). (F) Percentage of hyphae exhibiting tip accumulation of respective proteins (error bars, s.d., n = 3 independent experiments; > 100 hyphae per strain were analysed per experiment, ** p<0.01; *** p<0.001; ns, not significant, unpaired two-tailed t-test).

Normally, Kin3G^3^ (functional C-terminal Kin3-Gfp triple fusion) exhibits processive movement throughout hyphae. In the absence of Did2 processive signals were still visible, but the signal intensity of shuttling Kin3G signals was strongly reduced (Fig 5A; S5 Video). The reduction in signal intensity implies that fewer Kin3-molecules were loaded onto early endosomes. The fact that fewer Kin3 motors are present on the shuttling endosomes could very well explain why long-distance processive movement of endosomes is disturbed, since long range motility of endosomes in *U*. *maydis* is dependent upon Kin3 [25].

Cytoplasmic dynein transports endosomes from the poles towards the cell centre and in contrast to Kin3G^3^, dynein movement is independent of endosomal cargo [25]. Defects in microtubule-dependent transport of dynein results in its accumulation at the hyphal tip [48]. Analysing Dyn2G^3^ (functional C-terminal fusion of triple Gfp) revealed that without Did2 minus end-directed movement was still occurring, but Dyn2G^3^ exhibited stronger accumulations at the growth poles (Fig 5B–5D). This is indicative for alterations in dynein loading at the plus ends of microtubules for transport of endosomes away from the pole [46, 48]. Consistently, upon closer inspection we observed that a substantial amount of hyphae strongly accumulated Rab5a and Yup1CM positive signals at the hyphal tip (Fig 5E and 5F). Interestingly, these static accumulations did not contain comparably strong Rrm4 signals, indicating that the identity of this potential membranous compartment has changed (Fig 5E and 5F; S7 Fig). In essence, Did2 has a profound influence on the endosomal association of molecular motors, most likely by regulating the endosomal membrane composition. Consequently, processive long-distance movement of endosomes is disturbed.

### Did2 is needed for endosomal maturation

**Fig 6:**
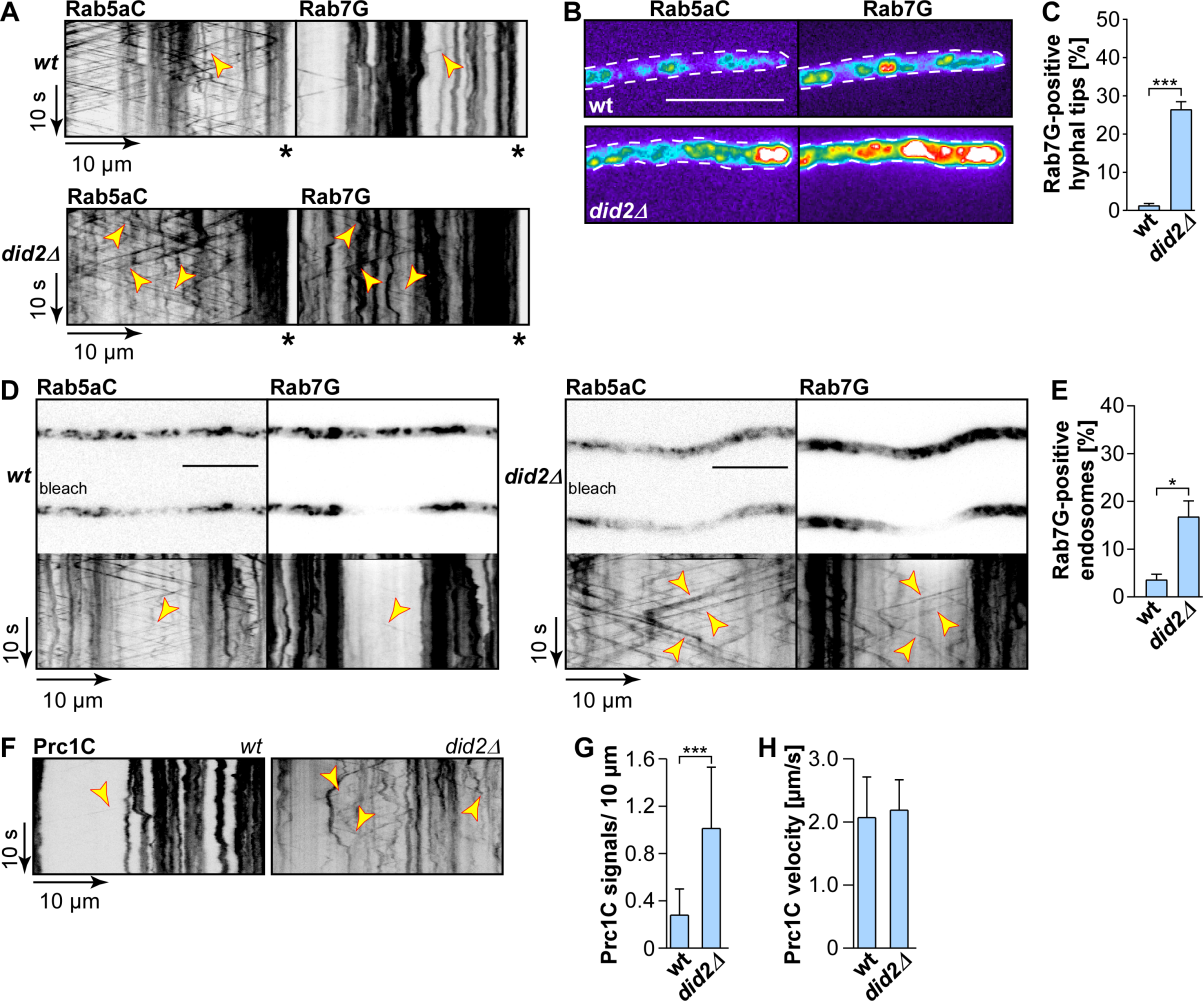
Endosomal maturation is disturbed in *did2Δ* hyphae. (A) Dynamic co-localisation studies of Rab5aC (left) and Rab7G (right). Fluorescence signals were detected simultaneously using dual colour imaging. Processive co-localizing signals are marked by arrowheads in the respective kymographs (hyphal tips are marked by asterisks). Strain with wildtype allele of *did2* (top) is compared to *did2Δ* strain (bottom). (B) Fluorescence micrograph in false colours (black/blue for low to red/white for high signal intensity) of strains expressing Rab5aC (left) and Rab7G (right) in the *did2* wildtype background (top) and *did2Δ* (bottom; size bar, 10 μm). (**C**) Bar chart depicting the percentage of Rab7-positive hyphal tips (error bars, s.e.m; n = 3 independent experiments; >100 hyphae per strain analysed per experiment; *** p<0.001 (unpaired two-tailed t-test). (D) Fluorescence micrographs of hyphae expressing Rab5aC and Rab7G before and after bleaching for improved detection of processive signals. Corresponding kymographs recorded after bleaching are shown below; wildtype allele of *did2* on the left and *did2Δ* on the right). Fluorescence signals of dynamic co-localisation studies were detected simultaneously using dual colour imaging (arrowheads indicate processive signals, size bar, 10 μm, S6 Video). (E) Bar chart depicting percentage of Rab7G-positive endosomes in the bleached area (error bars, s.d.; n = 3 independent experiments; 7 hyphae per strain were analysed per experiment); * p<0.05 (unpaired two-tailed t-test). (F) Kymographs of hyphae (6 h.p.i) expressing Prc1C in the *did2* wildtype background (left) and *did2Δ* (right); processive signals are marked by yellow arrowheads. Note that the empty area on the left corresponds to the nuclear region, which was chosen for better visibility of processive signals; hyphal tips are marked by asterisks; S7 Video). (G) Bar chart depicting amount of Prc1C signals per 10 μm hyphae (error bars, s.d.; N = 12 for wildtype and 14 for *did2Δ* hyphae were analysed, signals were scored as processive if run length > 5 μm; *** p<0.001 unpaired two-tailed t-test). (H) Bar chart depicting the velocity of processive signals (error bar, s.d.; number of hyphae given in G, corresponding to 30 signals in wildtype hyphae and more than 100 signals in *did2Δ* hyphae).

The reduced endosomal association of the molecular motors suggested a potentially altered membrane identity in *did2Δ* mutants. This prompted us to study markers of late endosomes such as Rab7, since it is known from *S*. *cerevisiae* that defects in the ESCRT complex results in disrupted endosomal maturation and hence aberrant accumulation of membranes [49]. Studying the subcellular localisation of Rab7G (Gfp fused N-terminally to Rab7, expressed ectopically at the *pep4*-locus) revealed that this protein localised mainly to static compartments such as late endosomes and vacuoles (S6C Fig) [19]. Thus, Rab7 marks a compartment, which is clearly distinct from Rab5a-positive structures (Fig 6A) [19]. However, Rab7 also occasionally shuttles on Rab5a-positive endosomes suggesting some overlap (Fig 6A). When focusing on the hyphal tip, loss of Did2 resulted in a drastic increase in Rab7G accumulations (Fig 6A–6C). Close to the growth pole Rab5aC and Rab7G signals co-localised extensively (Fig 6B) indicating that an aberrant compartment with altered membrane identity accumulated at the hyphal tip (Fig 5E).

Furthermore, loss of Did2 caused a significant increase of the processively moving subpopulation (Fig 6A). This was clearly observable when the background signal was reduced by photobleaching (Fig 6D and 6E; S6 Video). Hence, in the absence of Did2, shuttling of Rab7G on Rab5aC-positive endosomes increased, showing that more Rab7 is being aberrantly retained on early endosomes. This strongly suggests that endosomal maturation is disturbed.

Endosomal maturation is a requirement for sorting cargo towards the vacuole. We therefore revisited vacuolar targeting of the cargo protein Prc1 in hyphae. Prc1C-positive signals, i.e. vacuoles, were mainly static and distributed throughout hyphae. Loss of Did2 decreased the signal intensity of static Prc1C accumulation and caused an increase in processive movement, which was comparable to Rab7G (Fig 6F–6H; S7 Video). The velocity of shuttling Prc1 signals indicates that these signals represent endosomes (Fig 6H). Overall, the deletion of *did2* seems to impede the transition from motile early endosomes to static late endosomes, which strongly argues for the presence of an endosomal maturation defect in *did2Δ* hyphae. Such a defect would cause alterations of endosomal identity and thereby potentially disturb recruitment of endosomal proteins.

To summarise, in addition to the known role of Did2 in vacuolar trafficking, the ESCRT regulator is also crucial in determining the membrane identity of early endosomes in polar cells. The precise membrane identity of early endosomes guarantees their correct function in long-distance transport of other important cargos such as mRNAs and septins.

## Discussion

**Fig 7:**
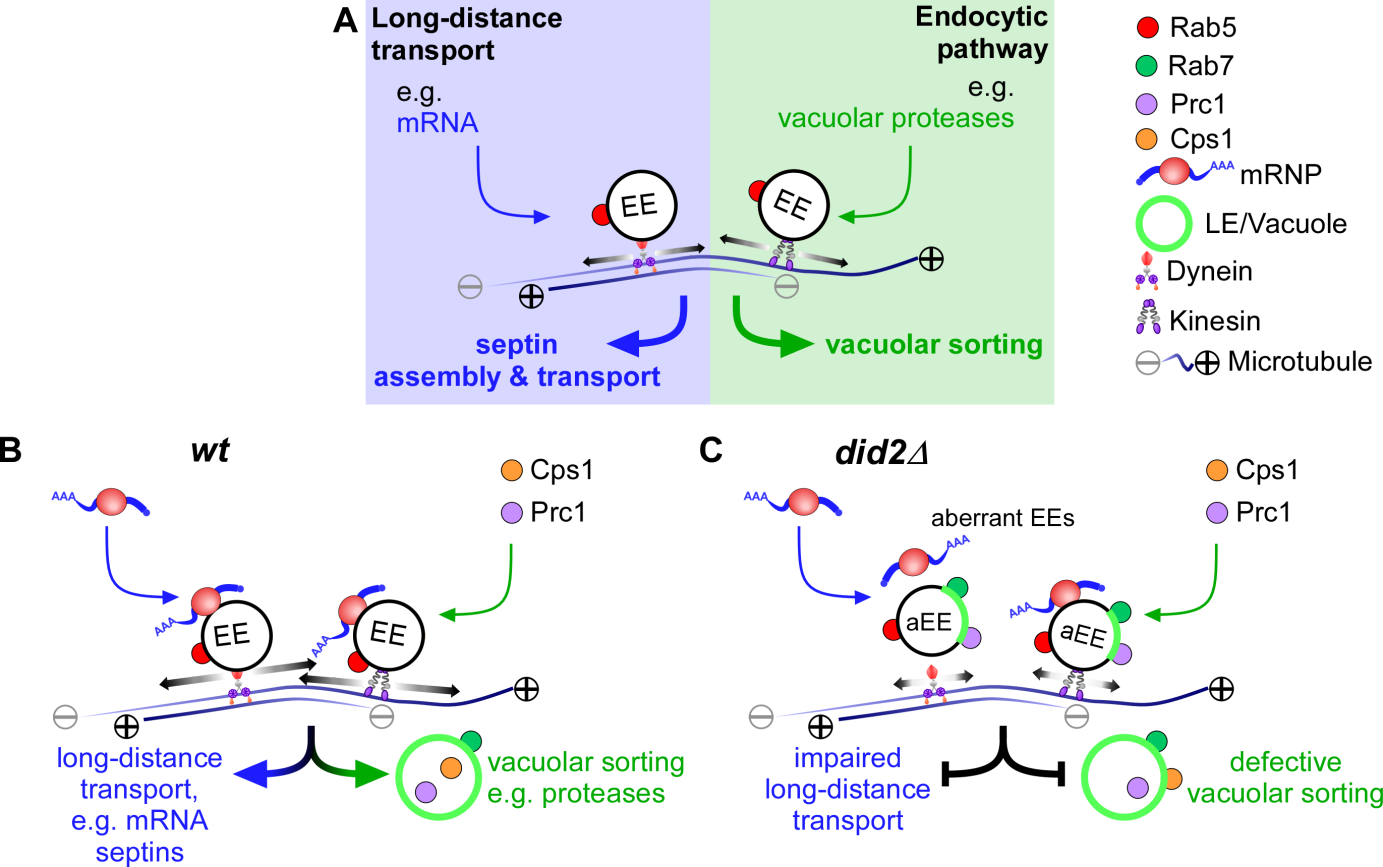
Did2 coordinates endosomal long-distance transport and endocytic trafficking. (A) The model at the top depicts two seemingly independent main functions of early endosomes (EE) that shuttle along microtubules. Long-distance transport involved in mRNA and septin transport on the left is compared to endocytic trafficking of e.g. vacuolar proteases on the right. At the bottom endosomal transport in the wildtype (B) is compared to transport events in the absence of ESCRT regulator Did2 (C). Note that loss of Did2 causes defects in maturation and changes the membrane identity (green area of EE). Therefore, Rab7 as well as cargo protein Prc1 is present more extensively on aberrant EEs, see text for further details.

In extended, highly polarised cells, like fungal hyphae, endosomes carry out at least two distinct functions. Firstly, during endocytosis they serve as transport vessels for protein cargo destined for degradation in the vacuole. Secondly, endosomes function in long-distance transport as a universal transport platform for proteins, organelles and mRNAs (Fig 7A) [28, 30, 50]. Thus far it was unclear how these two seemingly independent functions are interconnected and coordinated. By studying the ESCRT regulator Did2 we provide evidence, that the ESCRT machinery is important to orchestrate endocytic trafficking and long-distance transport.

### The ESCRT regulator Did2 is crucial for microtubule-dependent transport of early endosomes

Did2 is well-known for its regulation of the ESCRT component Vps4 and its function in endocytosis [10]. ESCRT dysfunction results in defects in endosomal maturation and protein sorting towards vacuoles/lysosomes. Consistently, deletion of *did2* causes similar defects in vacuolar protein sorting in *S*. *cerevisiae*, *A*. *nidulans* and *U*. *maydis* (this study), which underlines the conservation of this process in fungi [10, 44]. In filamentous fungi like *A*. *nidulans*, the absence of Did2 triggers aberrant hyphal growth, which correlates with altered recruitment of Rab5 effectors such as the PI3P kinase Vps34 to endosomes [51, 52]. Furthermore, loss of endosomal movement results in accumulation of vacuoles at the hyphal tip [53]. Thus, similar to the directionality in mammalian cells [54], active movement from the hyphal tip towards the basal pole appears to be crucial for the proper maturation from early to late endosomes (Fig 7A, right) [53, 55]. Consistently, it was shown that ESCRT components were present on shuttling Rab5-positive endosomes in *A*. *nidulans*, similar to the observations in this study [44]. Thus, components required for endosomal maturation such as ESCRT components and Rab GTPases are already present early on during long-distance trafficking [51, 52, 55].

Here, we discover that Did2 is not only needed for endocytic functions such as vacuolar sorting of cargo proteins (Fig 7A, right) but also essential for efficient long-distance transport (Fig 7A, left). Loss of Did2 caused reduced localisation of endosomal proteins and decreased processivity of endosomes as a whole. The observed endosome motility defects can be explained by the disturbed localisation of motor proteins such as Kin3, which is needed for processive movement along microtubules [25]. In addition, attachment of cytoplasmic dynein to early endosomes is most likely disturbed, which explains the accumulation of dynein at the plus-ends of microtubules, e. g. at the growing pole [48]. Overall, these observations point towards an altered membrane composition of early endosomes.

Endosomal maturation as well as fusion with vacuoles are dependent on a functional ESCRT machinery for vacuolar sorting. Consistently, we observe that in the absence of Did2 the late endosomal marker Rab7 as well as cargo protein vacuolar protease Prc1 shuttle more intensively on early endosomes, a phenomenon that is rarely seen in wildtype hyphae. This is consistent with current views that the first step during endosomal maturation is the formation of a Rab7-positive domain resulting in hybrid Rab5/Rab7-positive endosomes [56, 57]. One possible underlying mechanism is Rab conversion, by which Rab5 is replaced by Rab7 as the endosome matures [56, 58]. Alternatively, Rab7-positive domains destined for protein degradation could be sorted away via endosomal fission [57, 59, 60]. The latter explains why in ESCRT mutants of *S*. *cerevisiae* an aberrantly enlarged membrane compartment is formed (class E compartment) [49]. Moreover, the ESCRT regulator Ist1 was needed for endosomal fission in mammalian cells [61]. Consistently, the deletion of *rab7* (*rabS*) in *A*. *nidulans* induced the formation of motile “mini-vacuoles”, i.e. membrane compartments disturbed in maturation [53]. Similarly, downregulation of Rab7 in mammalian cells blocks cargo delivery from late endosomes to lysosomes [62]. Based on these results we propose that the formation of Rab7- and Prc1-positive shuttling endosomes in the absence of Did2 is due to disturbed fission of Rab7-positive lipid domains and their blocked fusion with vacuoles (Fig 7C). Consequently, dysfunctional maturation alters the identity of endosomes and thereby impairs the recruitment of endosomal proteins required for long-distance transport, like the FYVE-domain-containing mRNP linker protein Upa1 [29]. Thereby, endosomal movement of Rrm4 is disturbed resulting in altered transport of septin mRNAs and proteins [18, 20].

### The ESCRT regulator Did2 plays an indirect role in membrane-coupled mRNA transport

It is an emerging theme that mRNA transport and membrane trafficking are closely linked [26]. During oogenesis in *Drosophila melanogaster*, the small G protein Rab11 is important for *oskar* mRNA localisation [63] and loss of ESCRT-II components interfered with *bicoid* mRNA localisation during oogenesis. The RNA localisation element in *bicoid* mRNA might specifically be recognised by the ESCRT-II component Vps36 [64].

In this study, we identified mRNAs encoding the ESCRT-III regulators Did2 and Vps60 as potential targets of the mRNA transport machinery using pilot *in vivo* UV cross-linking iCLIP experiments [65]. A hallmark for mRNA transport is its link to protein localisation [66, 67]. Since Rrm4 binds numerous mRNA targets [31], it is conceivable that one function of endosomal mRNA transport might be the equal distribution of mRNAs to avoid artificial subcellular gradients. Interestingly, loss of Did2 and Rrm4 caused similar mutant phenotypes during hyphal growth supporting the initial hypothesis that Rrm4 regulates the subcellular localisation of Did2. Consistently, Did2 was also present on Rrm4-positive early endosomes. However, in contrast to other known targets of Rrm4 such as endosomal septins [18, 20], the subcellular localisation of Did2 was independent of Rrm4. Hence, unexpectedly, the Rrm4 machinery is not directly involved in determining the subcellular localisation of Did2. The identification of *did2* mRNA during *in vivo* UV crosslinking might be due to the fact that this technique is very sensitive and also identifies transient interactions [65].

Did2 was needed for correct endosomal localisation and processive movement of Rrm4 (see above). The altered localisation of Rrm4 coincided with impaired endosomal localisation of downstream components such as the poly(A)-binding protein and septins. Consequently, the formation of the septin gradient at the growing hyphal tip was reduced [17, 18, 20]. The most likely explanation for our observation of disturbed mRNA transport is the almost complete loss of the endosomal localisation of the adaptor protein Upa1 [29]. In essence, we discovered that there is a molecular link between ESCRT complex and endosomal mRNA transport. However, in this case it is not a direct link like proposed for *D*. *melanogaster* [64] but rather we observed an indirect role affecting loading of mRNPs on endosomes.

### Conclusion

Endosomal maturation is an important cellular process and endocytic recycling is required for extensive polar growth as observed in highly polarised cells such as neurons or fungal hyphae [22]. Studying the ESCRT regulator Did2 in *U*. *maydis* revealed that this protein is needed to coordinate and potentially insulate endocytic recycling from long-distance transport of endosomes (Fig 7B and 7C).

Interestingly, in highly polarised neuronal cells, there is increasing evidence for a close connection between ESCRT dysfunction, altered intracellular membrane trafficking and neuronal disease [68, 69]. Thus, it is conceivable that at least in part the altered processes are caused by disturbed long-distance transport. This is supported by recent findings studying axon elongation, where Rab5-mediated control of endosome trafficking is important for axon growth [70] and ESCRT-II transports mRNAs to regulate local protein synthesis [71]. In summary, our findings not only have implications for fungal biology but also provide new inspiration for neuronal research.

## Materials and methods

### Plasmids, strains, and growth conditions

Cloning was done using *E*. *coli* K-12 derivate Top10 (Life Technologies, Carlsbad, CA, USA). For *U*. *maydis*, growth conditions and antibiotics were described elsewhere [72]. Strains were constructed by the transformation of cells with linearised plasmids (S1 Table). All homologous integration events were verified by Southern blot analysis [72]. For ectopic integration, plasmids were linearised with SspI and targeted to the *ip*^*S*^ locus [73] or the *pep4* locus [42]. Genomic DNA of wild-type strain UM521 (a1b1) was used as a template for PCR amplifications unless otherwise noted. Detailed information is given in Supplementary S2 to S4 Tables and plasmid sequences are available upon request. The accession numbers of *U*. *maydis* genes used in this study are: *did2* (UMAG_05607), *vps60* (UMAG_05282), *prc1* (UMAG_04641), *cps1* (UMAG_05897), *rrm4* (UMAG_10836), *upa1* (UMAG_12183), *pab1* (UMAG_03494), *cdc3* (UMAG_10503), *rab5a* (UMAG_10615), *yup1* (UMAG_05406), *vps27* (UMAG_03862), *vps4* (UMAG_01669), *kin3* (UMAG_06251), *dyn2* (UMAG_04372), *rab7* (UMAG_05511).

### Bioinformatic analysis

All amino acid sequences used in sequence comparison were retrieved from the NCBI Entrez-database. Accession numbers: *U*. *maydis* Did2 (XP_011391906.1) and Vps60 (XP_011392001.1); *Saccharomyces cerevisiae* Did2p (NP_012961.3) and Vps60p (NP_010774); *Homo sapiens* CHMP1A (Q9HD42) and CHMP5 (NP_057494.3); *Aspergillus nidulans* DidB (XP_682665.1) and Vps60 (XP_663236.1); *Drosophila melanogaster* CHMP1A (NP_649051.3) and CHMP5 (NP_648997.1). The comparison was done using BlastP [35], domain predictions with SMART [36], while alignments were generated using the program ClustalX (Version 2.0.12) [74].

### Cell wall stress and filamentous growth on solid media

For growth on solid media, cell suspensions were spotted onto the respective plates at an OD_600_ of 0.5. For the analysis of colony morphology, 2 μl cell suspension were spotted onto CM (1% glc) plates, while 5 μl were spotted onto NM (1% glc) plates to induce filamentation. Cell wall stress was analysed by spotting 1:5 serial dilutions (down to OD_600_ of 8 * 10^−4^). 2 μl of the serial dilutions were spotted onto CM-glc plates supplemented with 25 μm or 50 μm Calcofluor (Sigma-Aldrich; Taufkirchen, Germany). All plates were incubated for 48 h at 28°C. The set-up used to acquire images was described before [29].

### Fluorometric measurement of endochitinolytic activity

Measurement of extracellular Cts1-activity was done as described before [31]. Strains were grown in CM (1% glc) medium to an OD_600_ of 0.5 and were either measured directy or shifted to NM (1% glc) medium for 6 h to induce filamentation. In total, three independent biological experiments were performed.

### Microscopy and image processing

The microscope set-ups used were described before [24, 45, 75]. Dual-color imaging was carried out as described in detail before [24]. GFP and RFP or mCherry fluorescence were simultaneously detected using a two-channel imager (DV2, Photometrics, Tucson, AZ). All images and videos were acquired and analysed using Metamorph (Versions 7.7.0.0 and 7.7.4.0, Molecular Devices, Sunnyvale, CA, USA). Strains were cultivated in 20 ml CM (1% glc) and grown to an OD_600_ of 0.5. Filamentous growth was induced by shifting cultures to NM (1% glc) for 4, 6 or 9 h at 28°C.

Cell division defects were quantified by scoring for the presence of cell aggregates. Since the *kin3Δ* strain forms large cell aggregates, aggregates were counted as single individuals. Cell length was assessed by measuring the length of single cells from pole-to-pole and in dividing cells from pole-to-septum of the mother cells. Bipolar growth of hyphae was assessed by scoring cells for unipolar, bipolar growth and septum formation. Tip accumulation was scored by acquiring z stacks (z distance 0.23 μm) and analyzing the maximum projections.

For the analysis of co-localisation, signal number, number of passages, and velocity of moving signals the acquired videos were converted to kymographs using Metamorph. Co-localisation was assessed by quantifying kymographs acquired by dual color imaging. Changes in direction were counted as individual signals. Signals were counted manually: processive signals (distance travelled > 5 μm), corralled movement (distance travelled < 5 μm) and static signals (distance travelled ~ 0 μm). Velocity was only measured for processive signals (movement >5 μm). For all quantifications, at least three independent experiments were analysed. Statistical analysis was performed using Prism5 (Graphpad, La Jolla, CA).

### FM4-64 and CMAC staining

FM4-64 staining of yeast and hyphal cells was done as described elsewhere [24]. For endosome labeling, 1 ml cell suspensions were labeled with 0.8 μM FM4-64 and incubated for 1 min at ambient temperature prior to microscopic analysis.

Vacuolar staining was performed using the dye CMAC (7-Amino-4-Chlorumethylcoumarin; Thermo Fisher Scientific, Darmstadt, Germany). 1 ml cell suspension was stained with 10 μM CMAC and incubated for 20 min at 28°C. Cells were washed once with PBS-buffer prior to microscopic analysis using a DAPI-filter set [75]. For co-labelling of vacuoles with FM4-64, cells were simultaneously stained with 10 μM CMAC and 0.8 μM FM4-64.

For tracking the uptake of FM4-64 over time, hyphal growth of AB33gfp and AB33did2Δ was induced by growth in NM for 6 hours. 500 μl culture of each strain were combined in a 2 ml tube and incubated for 10 min on ice. 4 μM (f.c.) FM4-64 was added and the cells were additionally incubated for 10 min on ice. Subsequently, cells were centrifuged, the cell pellet resuspended in 1 ml ice cold NM and then incubated on 28°C. FM4-64 uptake was tracked by fluorescence miscroscopy. Wildtypic AB33gfp cells could be distinguished from AB33did2Δ due to Gfp fluorescence.

### Photobleaching and photoactivation

Photoactivation and photobleaching were done using a 63x plan-apochromat objective (NA 1.4). For photoactivation, an area of 5 μm either in between nucleus and tip or 5 μm at the tip was activated by a 405 nm laser (40 mW), while the 488 nm laser (100 mW) for Gfp-excitation was set to 40%. For photobleaching, a region of 10 μm in between nucleus and hyphal tip was bleached by setting the 405 nm laser to 40 mW, and fluorescence was detected by dual color imaging (488 nm laser; Gfp-excitation): 20%; 561 nm laser (mCherry-excitation, 50%). Co-localisation within bleached region was determined by analyzing signals passing the middle of the bleached region.

## Supporting information

S1 Fig *vps60* and *did2* mRNAs are potential targets of Rrm4. (A) Schematic representation of preliminary *in vivo* UV cross-linking experiments following HITS-CLIP and iCLIP protocols [31, 76]. Open reading frames are represented as gray boxes, while the flanking untranslated regions (UTR) are drawn as thick black lines. UTRs were manually defined to be 300 nt in length. Potential crosslink sites are indicated as bars. (B-C) Sequence comparison of Did2 and Vps60 with orthologues from fungi and animals (accession numbers see Materials and Methods). Identical residues are highlighted in black. MIM1 motif is marked in red, while the MIM5 motif of Vps60 is highlighted in orange (region corresponds to *S*. *cerevisiae* Vps60p: aa 140–186) [33, 34].
(TIF)

S2 Fig Vps60 and Did2 are required for resistance against cell wall stress. (A) Growth of yeast cells in liquid cultures is shown by plotting the increase of optical density at 600 nm (OD_600_) over time (mean, s.e.m; n = 3 independent experiments). (B) Serial dilutions of strains were grown on plates in the absence or presence of calcofluor to induce cell wall stress. (C) Fluorescence micrographs (inverted pictures) of mixed cultures consisting of strains expressing Gfp carrying the wildtype alleles and strains carrying deletions of *vps60* (top) and *did2* (bottom). Vacuolar lumen was stained with CMAC. Endocytic trafficking was tested using FM4-64, a dye whose uptake follows the endocytic pathway (arrowheads indicate vacuoles, size bar, 10 μm). (D) Micrographs depicting wildtype and *did2Δ* strains 0 min (left) and 30 min (right) after giving a FM4-64 pulse (4 μM f.c.) to the growth medium. The images show FM4-64 fluorescence signal as an intensity heat map. Blue arrowheads indicate static vacuoles stained by FM4-64. (E) Kymographs showing the uptake of FM4-64 over time in AB33gfp (left) and AB33did2Δ (right) cells. The asterisk marks the hyphal tip. Yellow arrowheads indicate shuttling endosomes, whereas blue arrowheads indicate static vacuoles. Note, that the kymographs were generated from different hyphae.
(TIF)

S3 Fig Deletion of *did2* affects colony morphology. (A) Morphology of colonies grown on plates. (B) Micrograph of yeast cells dividing by budding. Defects in cell division lead to the formation of cell aggregates (asterisks; size bar, 10 μm). (C) Percentage of cells with defects in division (error bars, s.e.m.; at least 150 cells from three independent experiments were counted). (D) Length of cells (at least 150 cells per strain; shown are individual values and the medians as red lines.
(TIF)

S4 Fig Did2G does not rescue the mutant phenotype of *did2Δ* strain. (A) Morphology of colonies grown on plates. (B) Edges of colonies after induction of filamentous growth. (C) Micrographs of hyphae (6 h.p.i) of AB33 derivates (basal septa and growth direction are marked by asterisks and arrows, respectively; size bar, 10 μm). (D) Percentage of hyphae (6 h.p.i.): quantification of unipolarity, bipolarity and septum formation (mean, s.e.m.; n = 3 independent experiments, > 90 hyphae were counted per experiment). Note, that the *did2****Δ*** mutant phenotype can be complemented by ectopic expression of *did2*.
(TIF)

S5 Fig Did2G co-localizes with endosomal markers and loss of Did2 affects shuttling of Pab1 and the septin Cdc3. (A-B) Dynamic co-localisation studies of Did2G (left) with Rab5aC (A, right) and Rrm4C (B, right). Yellow arrowheads mark processive, co-localising signals (hyphal tips are marked by asterisks). (C-D) Kymographs of hyphae (6 h.p.i.) expressing Pab1G (C) and Cdc3G (D) (*did2* wildtype allele left and *did2****Δ*** right; processive signals are marked by yellow arrowheads; hyphal tips are marked by asterisks).
(TIF)

S6 Fig Processive movement of Rab5a-positive endosomes is reduced upon loss of Did2. (A) Fluorescence micrographs of strain expressing photoactivatable Rab5a-paG^3^ before and after photoactivation at the hyphal tip (red line). Corresponding kymographs are shown below (wildtype allele of *did2* top panel, *did2****Δ*** bottom panel). (B) Dynamic co-localisation studies of PhoxG (left) with FM4-64 (right). Fluorescence signals were detected simultaneously using dual colour imaging. Processive co-localizing signals are marked by yellow arrowheads (asterisks mark the hyphal tip). (C) Fluorescence micrographs (inverted images) of strains expressing Rab5aG, Yup1CM, PhoxG or Rab7G. Vacuolar lumen was stained with CMAC (right). Arrowheads mark vacuoles (arrows indicate growth direction; size bar, 10 μm). Not shown are initial cells of hyphae (4 h.p.i.), due to the better visibility of vacuoles in this hyphal region. (D-E) Dynamic co-localisation studies of Vps27G (D) and Vps4G (E) with FM4-64 as described in (B).
(TIF)

S7 Fig Rrm4 and Rab5a do not co-localise at the hyphal tip in the absence of Did2. Fluorescence micrograph in false colours (black/blue, low to red/white high) of strains expressing Rab5aC and Rrm4G (left and right respectively; *did2* wildtype allele top and *did2****Δ*** bottom; size bar, 10 μm). Dynamic co-localisation was performed with dual colour imaging.
(TIF)

S1 Table Description of *U*. *maydis* strains used in this study. (RTF)

S2 Table Generation of *U*. *maydis* strains used in this study. (RTF)

S3 Table Description of plasmids used for *U*. *maydis* strain generation. (RTF)

S4 Table DNA oligonucleotides used in this study. (RTF)

S1 Video Did2G moving bidirectionally and localizing in static accumulations in a hypha of AB33did2G. Video corresponds to Fig 2A (size bar, 10 μm, time in seconds, 150 ms exposure time, 150 frames, 5 frames/s display rate).
(MOV)

S2 Video Movement of Rrm4-Gfp in hyphae containing wildtype (wt; top) and *did2Δ* (bottom) allele. Deletion of *did2* disturbs movement of Rrm4G. The video corresponds to Fig 3A. Note, the kymograph shows only a subsection of this movie (size bar, 10 μm, time in seconds, 150 ms exposure time, 150 frames, 5 frames/s display rate).
(MOV)

S3 Video Movement of Rab5aG in hyphae containing the wt (top) and *did2Δ* (bottom) allele. Deletion of *did2* disturbs movement of Rab5aG. Video corresponds to Fig 4A. The kymographs show only a subsection of this movie (size bar, 10 μm, time in seconds, 150 ms exposure time, 150 frames, 5 frames/s display rate).
(MOV)

S4 Video Movement of photoactivatable Rab5a-paG^3^ after photoactivation in hyphae containing the wt (top) and *did2Δ* (bottom) allele. Deletion of *did2* reduces processive movement of Rab5a-paG^3^. Video corresponds to Fig 4E. Activated were regions of 5 μm in between the hyphal nucleus and tip (size bar, 10 μm, time in seconds, 150 ms exposure time, 150 frames, 5 frames/s display rate).
(MOV)

S5 Video Movement of Kin3G^3^ in hyphae containing the wt (top) and *did2Δ* (bottom) allele. Deletion of *did2* decreases the signal intensity of shuttling Kin3G^3^ signals. Examples of moving Kin3G^3^ particles are highlighted by arrowheads. The video corresponds to Fig 5A (size bar, 10 μm, time in seconds, 150 ms exposure time, 150 frames, 5 frames/s display rate).
(MP4)

S6 Video Dual color recording after photobleaching of Rab5a-mCherry and Rab7-Gfp in hyphae containing the wt (top) and *did2Δ* (bottom) allele. Dual color imaging of Rab5a-mCherry and Rab7-G after photobleaching with a 405 nm laser shows that localisation of Rab7G on moving endosomes increases upon deletion of *did2*. Moving signals are highlighted in the respective channel by arrowheads. The video corresponds to Fig 6D (size bar, 10 μm, time in seconds, 150 ms exposure time, 150 frames, 5 frames/s display rate).
(MP4)

S7 Video Intracellular localisation of Prc1-mCherry in hyphae containing the wt (top) and *did2Δ* (bottom) allele. Movement of Prc1C is increasingly observable upon deletion of *did2*. Note, the intensity of the vacuolar signal of Prc1C is decreased in *did2Δ* hyphae. The video corresponds to Fig 6F (size bar, 10 μm, time in seconds, 150 ms exposure time, 150 frames, 5 frames/s display rate).
(MOV)
