# Supplementary figures and images for: The ESCRT regulator Did2 maintains the balance between long-distance endosomal transport and endocytic trafficking
Source: PLoS Genet. 2017 Apr 19;13(4):e1006734. doi: 10.1371/journal.pgen.1006734 (PMC5415202; doi:10.1371/journal.pgen.1006734)

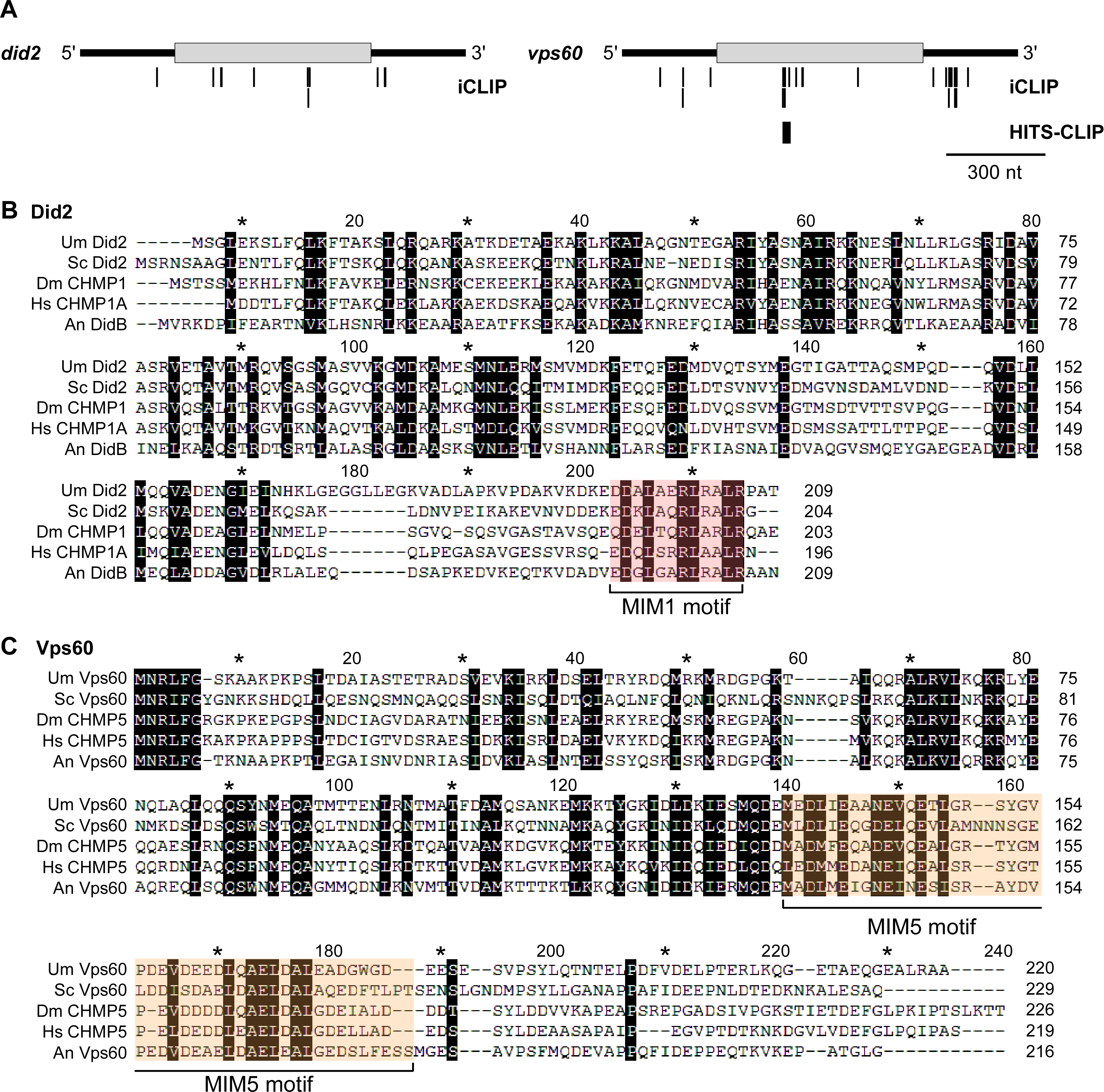

Supplement: S1 Fig — (A) Schematic representation of preliminary in vivo UV cross-linking experiments following HITS-CLIP and iCLIP protocols [31, 76]. Open reading frames are represented as gray boxes, while the flanking untranslated regions (UTR) are drawn as thick black lines. UTRs were manually defined to be 300 nt in length. Potential crosslink sites are indicated as bars. (B-C) Sequence comparison of Did2 and Vps60 with orthologues from fungi and animals (accession numbers see Materials and Methods). Identical residues are highlighted in black. MIM1 motif is marked in red, while the MIM5 motif of Vps60 is highlighted in orange (region corresponds to S. cerevisiae Vps60p: aa 140–186) [33, 34]. (TIF) [file pgen.1006734.s001.tif]

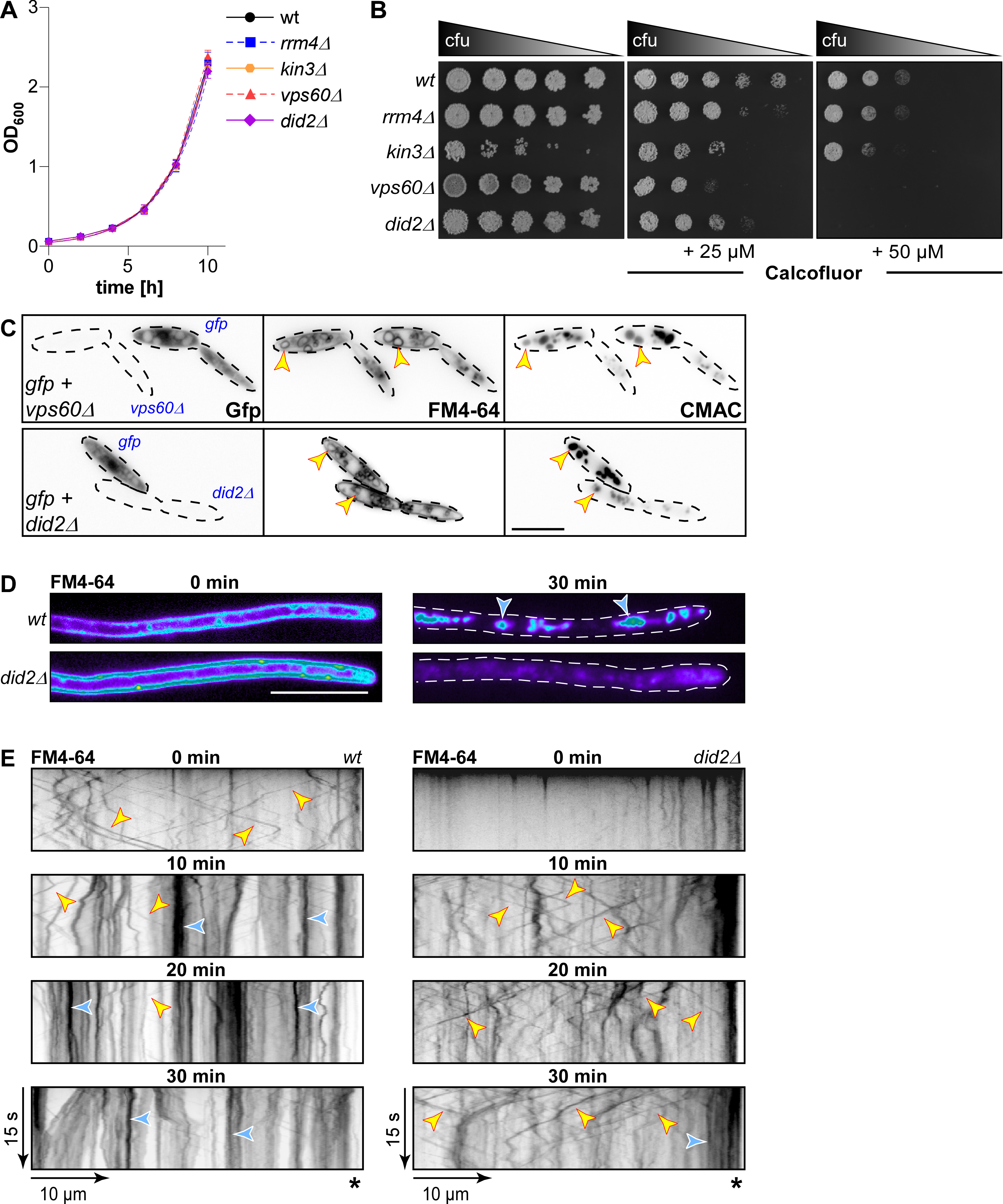

Supplement: S2 Fig — (A) Growth of yeast cells in liquid cultures is shown by plotting the increase of optical density at 600 nm (OD600) over time (mean, s.e.m; n = 3 independent experiments). (B) Serial dilutions of strains were grown on plates in the absence or presence of calcofluor to induce cell wall stress. (C) Fluorescence micrographs (inverted pictures) of mixed cultures consisting of strains expressing Gfp carrying the wildtype alleles and strains carrying deletions of vps60 (top) and did2 (bottom). Vacuolar lumen was stained with CMAC. Endocytic trafficking was tested using FM4-64, a dye whose uptake follows the endocytic pathway (arrowheads indicate vacuoles, size bar, 10 μm). (D) Micrographs depicting wildtype and did2Δ strains 0 min (left) and 30 min (right) after giving a FM4-64 pulse (4 μM f.c.) to the growth medium. The images show FM4-64 fluorescence signal as an intensity heat map. Blue arrowheads indicate static vacuoles stained by FM4-64. (E) Kymographs showing the uptake of FM4-64 over time in AB33gfp (left) and AB33did2Δ (right) cells. The asterisk marks the hyphal tip. Yellow arrowheads indicate shuttling endosomes, whereas blue arrowheads indicate static vacuoles. Note, that the kymographs were generated from different hyphae. (TIF) [file pgen.1006734.s002.tif]

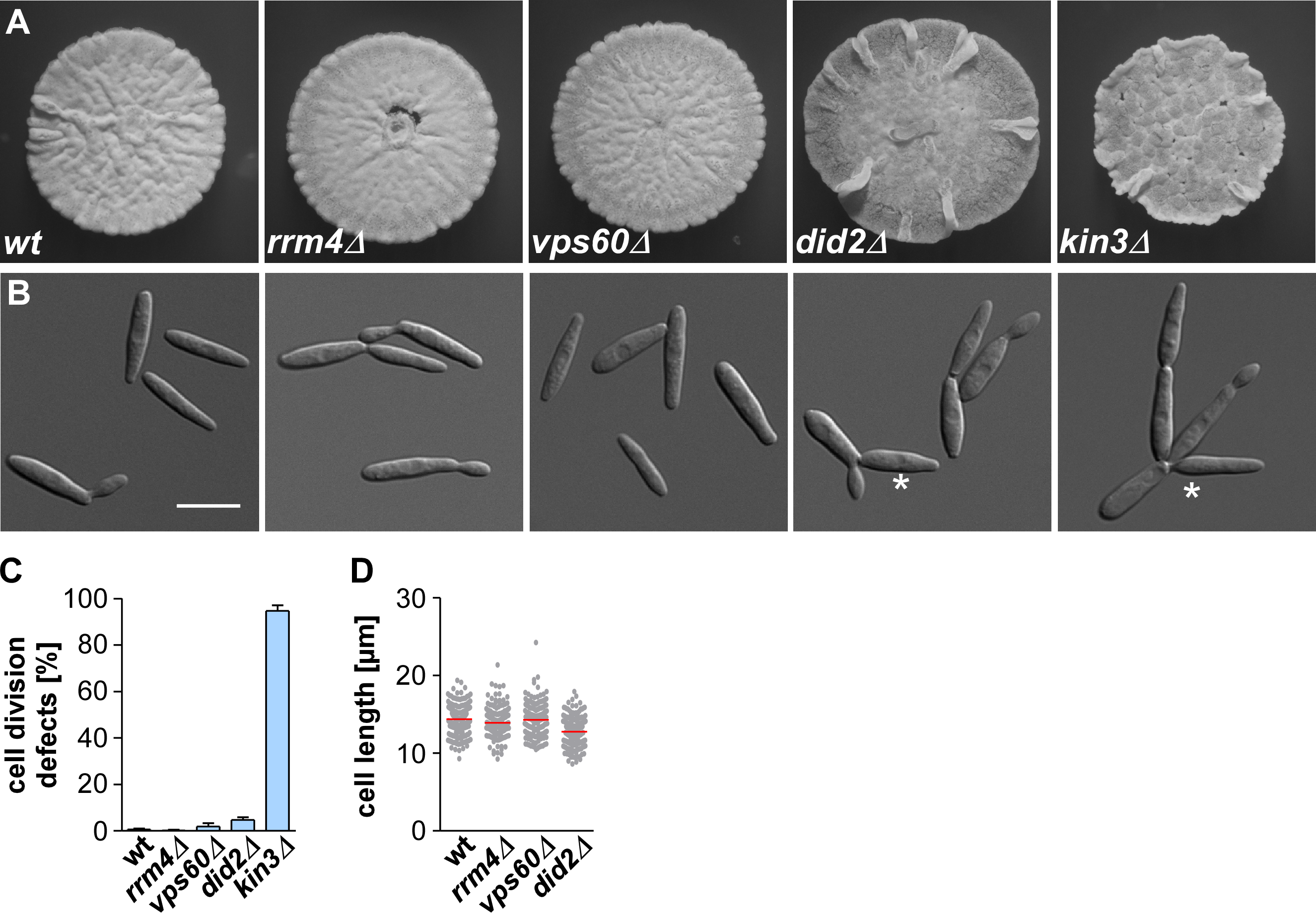

Supplement: S3 Fig — (A) Morphology of colonies grown on plates. (B) Micrograph of yeast cells dividing by budding. Defects in cell division lead to the formation of cell aggregates (asterisks; size bar, 10 μm). (C) Percentage of cells with defects in division (error bars, s.e.m.; at least 150 cells from three independent experiments were counted). (D) Length of cells (at least 150 cells per strain; shown are individual values and the medians as red lines. (TIF) [file pgen.1006734.s003.tif]

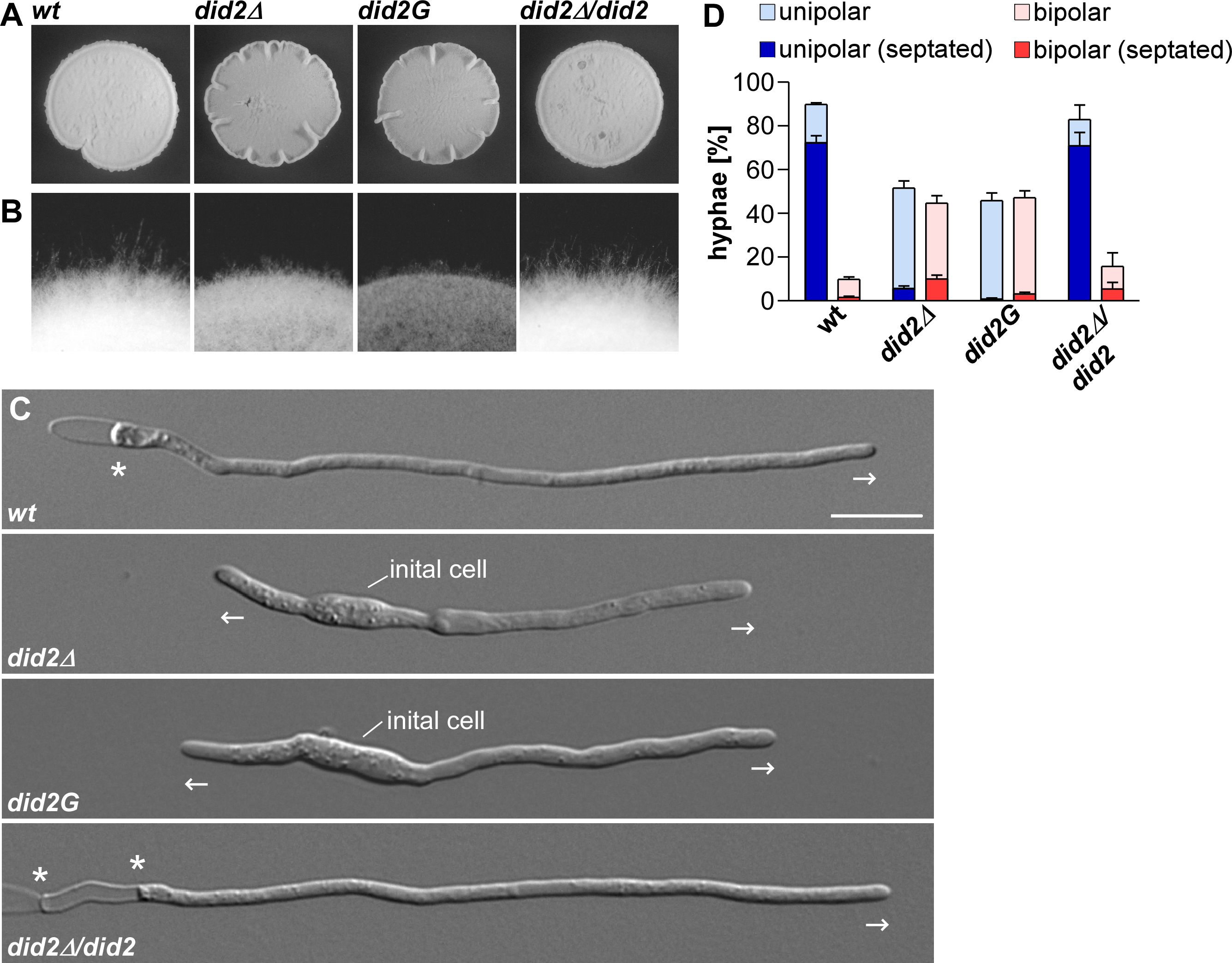

Supplement: S4 Fig — (A) Morphology of colonies grown on plates. (B) Edges of colonies after induction of filamentous growth. (C) Micrographs of hyphae (6 h.p.i) of AB33 derivates (basal septa and growth direction are marked by asterisks and arrows, respectively; size bar, 10 μm). (D) Percentage of hyphae (6 h.p.i.): quantification of unipolarity, bipolarity and septum formation (mean, s.e.m.; n = 3 independent experiments, > 90 hyphae were counted per experiment). Note, that the did2Δ mutant phenotype can be complemented by ectopic expression of did2. (TIF) [file pgen.1006734.s004.tif]

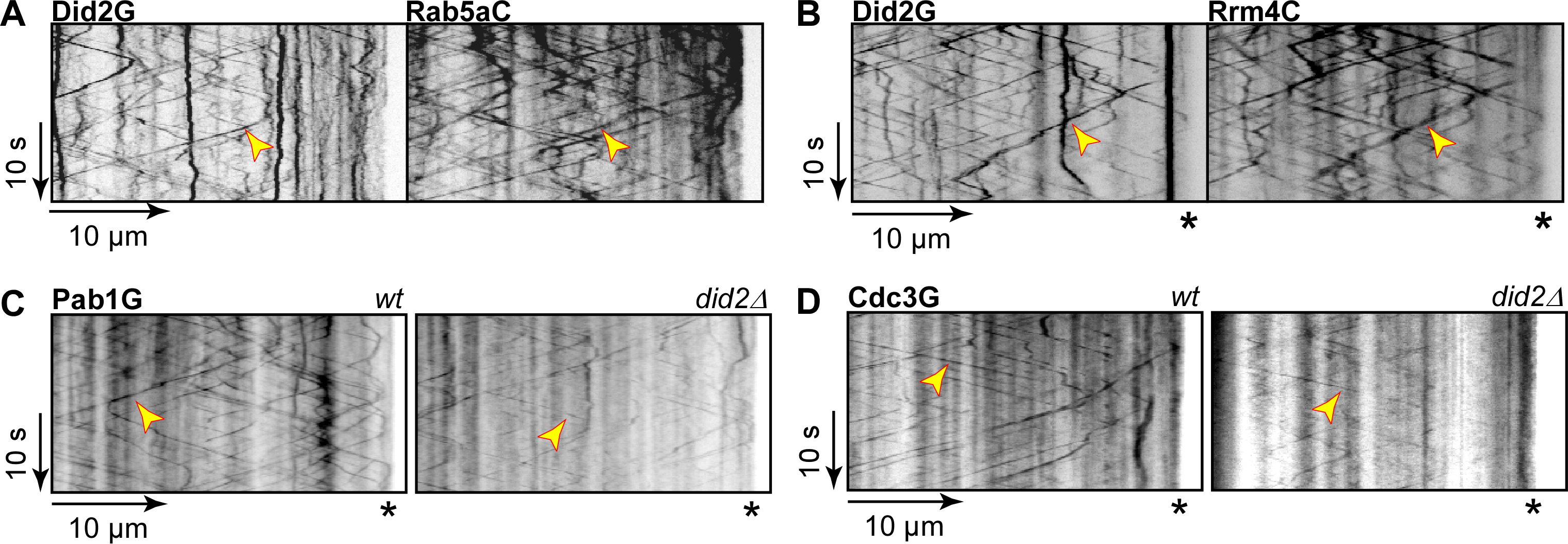

Supplement: S5 Fig — (A-B) Dynamic co-localisation studies of Did2G (left) with Rab5aC (A, right) and Rrm4C (B, right). Yellow arrowheads mark processive, co-localising signals (hyphal tips are marked by asterisks). (C-D) Kymographs of hyphae (6 h.p.i.) expressing Pab1G (C) and Cdc3G (D) (did2 wildtype allele left and did2Δ right; processive signals are marked by yellow arrowheads; hyphal tips are marked by asterisks). (TIF) [file pgen.1006734.s005.tif]

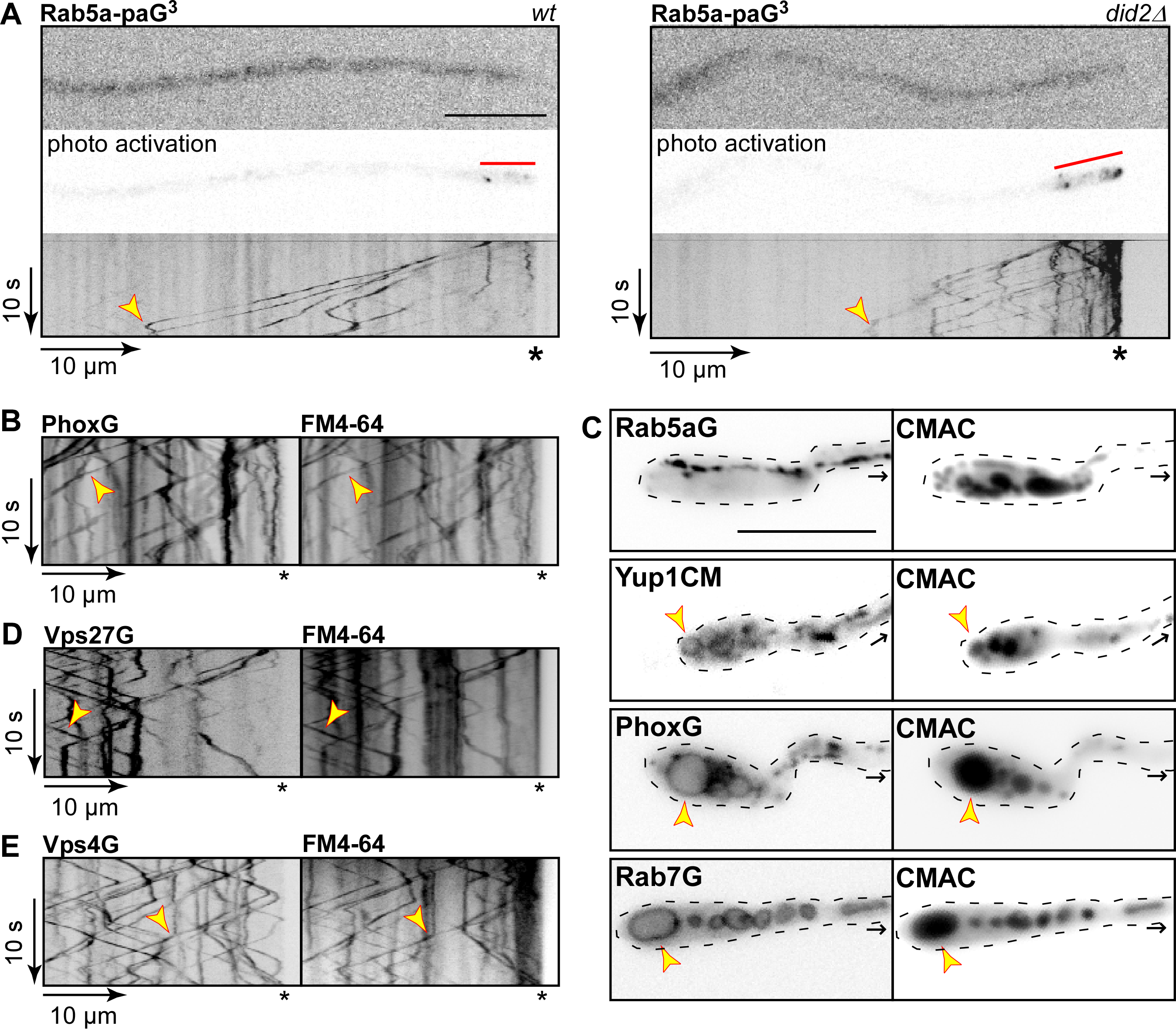

Supplement: S6 Fig — (A) Fluorescence micrographs of strain expressing photoactivatable Rab5a-paG3 before and after photoactivation at the hyphal tip (red line). Corresponding kymographs are shown below (wildtype allele of did2 top panel, did2Δ bottom panel). (B) Dynamic co-localisation studies of PhoxG (left) with FM4-64 (right). Fluorescence signals were detected simultaneously using dual colour imaging. Processive co-localizing signals are marked by yellow arrowheads (asterisks mark the hyphal tip). (C) Fluorescence micrographs (inverted images) of strains expressing Rab5aG, Yup1CM, PhoxG or Rab7G. Vacuolar lumen was stained with CMAC (right). Arrowheads mark vacuoles (arrows indicate growth direction; size bar, 10 μm). Not shown are initial cells of hyphae (4 h.p.i.), due to the better visibility of vacuoles in this hyphal region. (D-E) Dynamic co-localisation studies of Vps27G (D) and Vps4G (E) with FM4-64 as described in (B). (TIF) [file pgen.1006734.s006.tif]

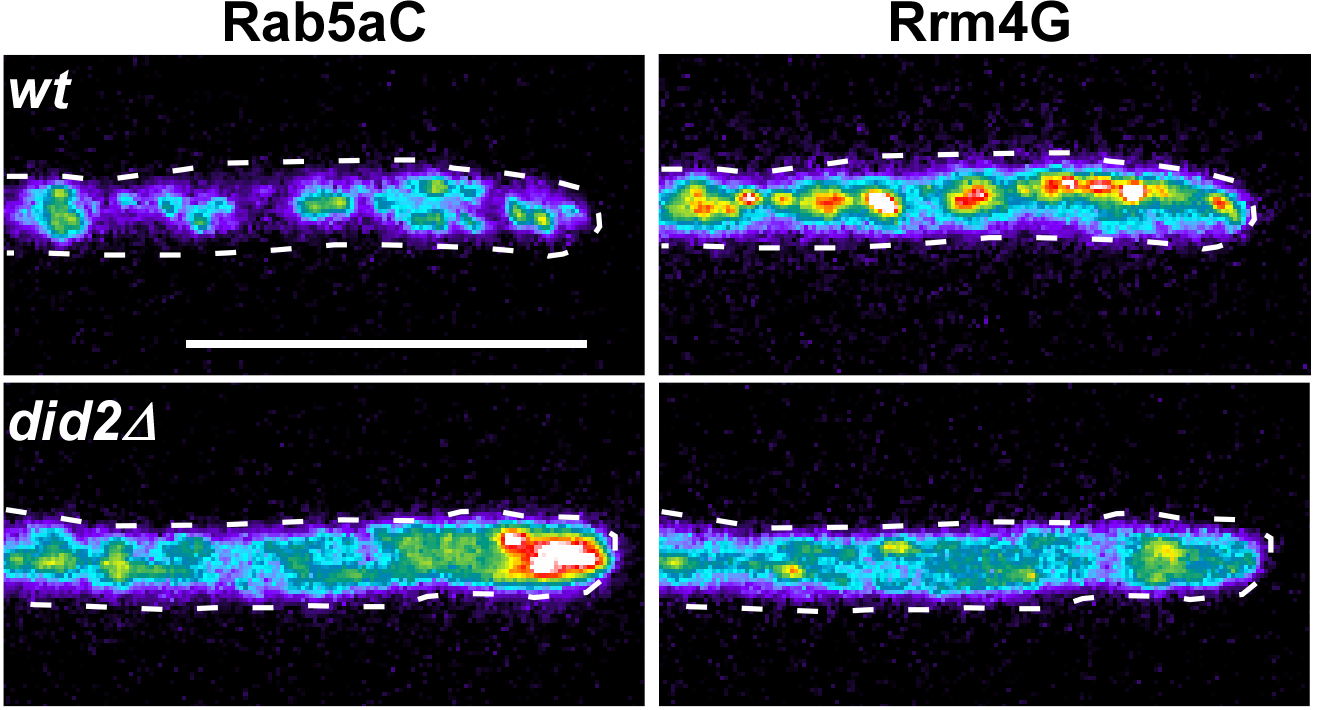

Supplement: S7 Fig — Fluorescence micrograph in false colours (black/blue, low to red/white high) of strains expressing Rab5aC and Rrm4G (left and right respectively; did2 wildtype allele top and did2Δ bottom; size bar, 10 μm). Dynamic co-localisation was performed with dual colour imaging. (TIF) [file pgen.1006734.s007.tif]
