# Supplementary material for: The ESCRT regulator Did2 maintains the balance between long-distance endosomal transport and endocytic trafficking
Source: PLoS Genet. 2017 Apr 19;13(4):e1006734. doi: 10.1371/journal.pgen.1006734 (PMC5415202; doi:10.1371/journal.pgen.1006734)
Supplement: S1 Table — (RTF) [file pgen.1006734.s008.rtf]

S1 Table: Description of U. maydis strains used in this study
Strain	Relevant genotype	Short description	
AB33	a2 Pnar:bW2 bE1	Transcription of the bE/bW genes is under control of the inducible Pnar-promotor. Filamentous growth can be induced by switching the nitrogen source.	
AB33 rrm4D	rrm4D	Carries rrm4 deletion	
AB33 kin3D	kin3D	Carries kin3 deletion	
AB33 vps60D	vps60D	Carries vps60 deletion	
AB33 did2D	did2D	Carries did2 deletion	
AB33 did2G	did2G	Expresses Did2-Gfp and carries rrm4 deletion	
AB33 rrm4D/did2G	rrm4D/did2G	Expresses Did2-Gfp and carries rrm4 deletion	
AB33 did2G/rrm4C	rrm4C/did2G	Co-expresses Did2-Gfp and Rrm4-mCherry	
AB33 did2G/rab5C	rab5aC/did2G	Co-expresses Did2-Gfp and mCherry-Rab5a under control of the consitutive Ptef-promotor. The construct is ectopically integrated at the ipS locus.	
AB33 cdc3G	cdc3G	Expresses Gfp-Cdc3	
AB33 cdc3G/rrm4D	cdc3G/rrm4D	Expresses Gfp-Cdc3 and carries a rrm4 deletion	
AB33 cdc3G/did2D	cdc3G/did2D	Expresses Gfp-Cdc3 and carries a did2 deletion	
AB33 rrm4G	rrm4G	Expresses Rrm4-Gfp	
AB33 rrm4G/did2D	rrm4G/did2D	Expresses Rrm4-Gfp and carries a did2 deletion	
AB33 pab1G	pab1G	Expresses Pab1-Gfp	
AB33 pab1G/did2D	pab1G/did2D	Expresses Pab1-Gfp and carries a did2 deletion	
AB33 upa1G	upa1G	Expresses Upa1-Gfp	
AB33 upa1G/did2D	upa1G/did2D	Expresses Upa1-Gfp and carries a did2 deletion	
AB33 rab5aG	rab5aG	Expresses Gfp-Rab5a under control of the strong constitutive Potef-Promotor. The Rab5a construct is ectopically integrated at the ips locus	
AB33 rab5aG/did2D	rab5aG/did2D	Expresses Gfp-Rab5a under control of the strong constitutive Potef-Promotor and carries a did2 deletion. The Rab5a construct is ectopically integrated at the ips locus	
AB33 rab5a-paG3n	rab5a-paG3	Expresses photoactivateable paGfp3-Rab5a under control of the strong constitutive Potef-promotor. The Rab5a construct is ectopically integrated at the ips locus.	
AB33 did2D/Rab5a-paG3	did2D/rab5a-paG3	Expresses photoactivateable paGfp3-Rab5a under control of the strong constitutive Potef-promotor. The Rab5a construct is integrated ectopically into the ips locus. The strain also carries a did2 deletion.	
AB33 rrm4G/rab5aC	rrm4G/rab5aC	Co-expresses Rrm4-Gfp and mCherry-Rab5a. The Rab5a construct is under control of the constitutive Ptef-promotor and ectopically integrated at the ips locus.	
AB33 rrm4G/rab5aC/upa1D	rrm4G/rab5aC/upa1D	Co-expresses Rrm4-Gfp, mCherry-Rab5a, and carrying a upa1 deletion. The Rab5a construct is under control of the constitutive Ptef-promotor and ectopically integrated at the ips locus.	
AB33 rrm4G/rab5aC/did2D	rrm4G/rab5aC/did2D	Co-expresses Rrm4-Gfp, mCherry-Rab5a and carries a did2 deletion. The Rab5a construct is under control of the constitutive Ptef promotor and ectopically integrated at the ips locus.	
AB33 yup1CM	yup1CM	Expresses Yup1-mCherry-3x myc under control of the strong constitutive Potef-promotor. The Yup1 construct is ectopically integrated at the ips locus.	
AB33 yup1CM/did2D	yup1CM/did2D	Expresses Yup1-mCherry 3x myc under control of the strong constitutive Potef-promotor and carries a did2 deletion. The Yup1 construct is ectopically integrated at the ips locus.	
AB33 phoxG	phoxG	Expresses the Phox domain of Yup1 (aa 1-148) fused C-terminally to Gfp. Phox-Gfp is under control of the strong constitutive Potef pronotor and is ectopically integrated at the ips locus.	
AB33 did2D/phoxG	did2D/phoxG	Expresses the Phox domain of Yup1 (aa 1-148) fused C-terminally to Gfp and carries a did2 deletion. Phox-Gfp is under control of the strong constitutive Potef pronotor and is ectopically integrated at the ips locus.	
AB33 vps27G	vps27G	Expresses Vps27-Gfp	
AB33 vps27G/did2D	vps27G/did2D	Expresses Vps27-Gfp and carries did2 deletion	
AB33 vps4G	vps4G	Expresses Vps4-Gfp	
AB33 vps4G/did2D	vps4G/did2D	Expresses Vps4-Gfp and carries did2 deletion	
AB33 kin3G3	kin3G3	Expresses Kin3-Gfp3	
AB33 kin3G3/did2D	kin3G3/did2D	Expresses Kin3-Gfp3 and carries did2 deletion	
AB33 dyn2G3	dyn2G3	Expresses Dyn2-Gfp3	
AB33 dyn2G3/did2D	dyn2G3/did2D	Expresses Dyn2-Gfp3 and carries did2 deletion	
AB33 rab5aC/rab7G	rab5aC/rab7G/pep4D	Co-expresses mCherry-Rab5a, Gfp-Rab7, and carrying a pep4 deletion. Both constructs are ectopically inserted into the genome and under the control of constitutive promotors: Ptef for Rab5aC (ips locus) and Po2tef for Rab7G (pep4-locus)	
AB33rab5C/did2D/rab7G	rab5aC/did2D/rab7G/pep4D	Co-expresses mCherry-Rab5a, Gfp-Rab7, and carries pep4 & did2 deletions. Both constructs are ectopically inserted into the genome and under the control of constitutive promotors: Ptef for Rab5aC (ips locus) and Po2tef for Rab7G (pep4 locus)	
AB33 prc1C	prc1C	Expresses Prc1-mCherry under control of the strong constitutive Potef-promotor. The construct is ectopically inserted into the ips locus.	
AB33 did2D/prc1C	did2D/prcC1	Carries a did2 deletion and expresses Prc1-mCherry under control of the strong constitutive Potef-promotor. The construct is ectopically inserted into the ips locus.	
AB33 vps60D/prc1C	vps60D/prc1	Carries a vps60 deletion and expresses Prc1-mCherry under control of the strong constitutive Potef-promotor. The construct is ectopically inserted into the ips locus.	
AB33 cps1C	cps1C	Expresses mChery-Cps1 under control of the constitutive Ptef-promotor. The construct is ectopically inserted into the ips locus.	
AB33 did2D/cps1C	did2D/cps1C	Carries a did2 deletion and expresses mChery-Cps1 under control of the constitutive Ptef-promotor. The construct is ectopically inserted into the ips locus.	
AB33 vps60D/cps1C	vps60D/cps1C	Carries a vps60 deletion and expresses mChery-Cps1 under control of the constitutive Ptef-promotor. The construct is ectopically inserted into the ips locus.	
AB33 did2D/did2	did2D/did2	Carries a did2 deletion and expresses ectopically did2 under control of the constitutive Ptef-promotor. The ectopic copy of did2 is inserted into the ips locus.	
