# Supplementary material for: The ESCRT regulator Did2 maintains the balance between long-distance endosomal transport and endocytic trafficking
Source: PLoS Genet. 2017 Apr 19;13(4):e1006734. doi: 10.1371/journal.pgen.1006734 (PMC5415202; doi:10.1371/journal.pgen.1006734)
Supplement: S2 Table — (RTF) [file pgen.1006734.s009.rtf]

S2 Table: Generation of U. maydis strains used in this study
Strain	Relevant genotype	UMa	Reference	Transformed plasmid	Locus	Progenitor	
AB33	a2 Pnar:bW2 bE1	133	Brachmann et al., 2001	pAB33	b	FB2	
AB33 rrm4D	rrm4D	273	Becht et al., 2006	pRrm4D-HygR (pUMa495)	rrm4	AB33	
AB33 kin3D	kin3D	662	Baumann et al., 2012	pKin3D-CbxR (pUMa1231)	kin3	AB33	
AB33 vps60D	vps60D	933	this study	pVps60D-HygR (pUMa1698)	vps60	AB33	
AB33 did2D	did2D	934	this study	pDid2D-HygR (pUMa1699)	did2	AB33	
AB33 did2G	did2G	1149	this study	pDid2G-NatR (pUMa1700)	did2	AB33 did2D	
AB33 rrm4D/did2G	rrm4D/did2G	1148	this study	pDid2G-NatR (pUMa1700)	did2	AB33 rrm4D	
AB33 did2G/rrm4C	rrm4C/did2G	1219	this study	pDid2G-NatR (pUMa1700)	did2	AB33 rrm4-Cherry	
AB33 did2G/rab5C	rab5aC/did2G	1725	this study	pDid2G-NatR (pUMa1700)	did2	AB33 rab5a-Cherry	
AB33 cdc3G	cdc3G	449	Baumann et al., 2014	pCdc3G-NatR (pUMa1028)	cdc3	AB33 cdc3D	
AB33 cdc3G/rrm4D	cdc3G/rrm4D	462	Baumann et al., 2014	PCR-fragment (rrm4D-HygR)	rrm4	AB33 cdc3G	
AB33 cdc3G/did2D	cdc3G/did2D	1480	this study	pDid2D-HygR (pUMa1699)	did2	AB33 cdc3G	
AB33 rrm4G	rrm4G	274	Becht et al., 2006	pRrm4G-NatR (pUMa496)	rrm4	AB33rrm4D	
AB33 rrm4G/did2D	rrm4G/did2D	1174	this study	pDid2D-HygR (pUMa1699)	did2	AB33 rrm4G	
AB33 pab1G	pab1G	389	König et al., 2009	pPab1G-NatR (pUMa805)	pab1	AB33	
AB33 pab1G/did2D	pab1G/did2D	1480	this study	pDid2D-HygR (pUMa1699)	did2	AB33 pab1G	
AB33 upa1G	upa1G	956	Pohlmann et al.,2015	pUpa1G-NatR (pUMa1575)	upa1	AB33	
AB33 upa1G/did2D	upa1G/did2D	1552	this study	pDid2D-HygR (pUMa1699)	did2	AB33 upa1G	
AB33 rab5aG	rab5aG	826	Baumann et al., 2012	pRab5aG-CbxR
(pUMa1481)	ipS	AB33	
AB33 rab5aG/did2D	rab5aG/did2D	998	this study	pDid2D-HygR (pUMa1699)	did2	AB33 rab5aG	
AB33 rab5a-paG3n	rab5a-paG3	1500	this study	pRab5a-paG3-CbxR (pUMa1477)	ipS	AB33	
AB33 did2D/Rab5a-paG3	did2D/rab5a-paG3	1130	this study	pRab5a-paG3-CbxR (pUMa1477)	ipS	AB33 did2D	
AB33 rrm4G/rab5aC	rrm4G/rab5aC	1053	Baumann et al., 2012	pRab5aC-CbxR (pUMa1806)	ipS	AB33 rrm4G	
AB33 rrm4G/rab5aC/upa1D	rrm4G/rab5aC/upa1D	1056	Pohlmann et al.,2015	pRab5aC-CbxR (pUMa1806)	ipS	AB33 rrm4G/upa1D	
AB33 rrm4G/rab5aC/did2D	rrm4G/rab5aC/did2D	1705	this study	pDid2D-HygR (pUMa1699)	did2	AB33 rrm4G/rab5aC	
AB33 yup1CM	yup1CM	685	Baumann et al., 2012	pYup1CM-CbxR (pUMa1376)	ipS	AB33	
AB33 yup1CM/did2D	yup1CM/did2D	1806	this study	pDid2D-HygR (pUMa1699)	did2	AB33 yup1CM	
AB33 phoxG	phoxG	1960	this study	pPhoxG-CbxR (pUMa2931)	ipS	AB33	
AB33 did2D/phoxG	did2D/phoxG	1961	this study	pPhoxG-CbxR (pUMa2931)	ipS	AB33 did2D	
AB33 vps27G	vps27G	935	this study	pVps27G-NatR (pUMa1714)	vps27	AB33	
AB33 vps27G/did2D	vps27G/did2D	1770	this study	pDid2D-G418R (pUMa2452)	did2	AB33 vps27G	
AB33 vps4G	vps4G	1568	this study	pVps4G-NatR (pUMa1871)	vps4	AB33	
AB33 vps4G/did2D	vps4G/did2D	1455	this study	pDid2D-HygR (pUMa1699)	did2	AB33 vps4G	
AB33 kin3G3	kin3G3	661	Baumann et al., 2012	pKin3G3-HygR (pUMa1295)	kin3	AB33	
AB33 kin3G3/did2D	kin3G3/did2D	1805	this study	pDid2D-G418R (pUMa2452)	did2	AB33 kin3G3	
AB33 dyn2G3	dyn2G3	1928	this study	pDyn2G3-HygR (pUMa965)	dyn2	AB33	
AB33 dyn2G3/did2D	dyn2G3/did2D	1929	this study	pDid2D-G418R (pUMa2452)	did2	AB33 did2D	
AB33 rab5aC/rab7G	rab5aC/rab7G/pep4D	1750	this study	pPep4D: Rab7G-NatR (pUMa2666)	pep4	AB33 rab5aC	
AB33rab5C/did2D/rab7G	rab5aC/did2D/rab7G/pep4D	1757	this study	pPep4D: Rab7Gn-NatR (pUMa2666)	pep4	AB33 rab5aC/did2D	
AB33 prc1C	prc1C	1250	this study	pPrc1C-CbxR (pUMa2135)	ipS	AB33	
AB33 did2D/prc1C	did2D/prc1C	1704	this study	pPrc1C-CbxR (pUMa2135)	ipS	AB33 did2D	
AB33 vps60D/prc1C	vps60D/prc1C	1703	this study	pPrc1C-CbxR (pUMa2135)	ipS	AB33vps60D	
AB33 cps1C	cps1C	1769	this study	pCps1C-CbxR (pUMa2743)	ipS	AB33	
AB33 did2D/cps1C	did2D/cps1C	1771	this study	pCps1C-CbxR (pUMa2743)	ipS	AB33 did2D	
AB33 vps60D/cps1C	vps60D/cps1C	1772	this study	pCps1C-CbxR (pUMa2743)	ipS	AB33 vps60D	
AB33 did2D/did2	did2D/did2	1801	this study	pDid2-CbxR (pUMa2710)	ipS	AB33 did2D	
