# Supplementary material for: The ESCRT regulator Did2 maintains the balance between long-distance endosomal transport and endocytic trafficking
Source: PLoS Genet. 2017 Apr 19;13(4):e1006734. doi: 10.1371/journal.pgen.1006734 (PMC5415202; doi:10.1371/journal.pgen.1006734)
Supplement: S3 Table — (RTF) [file pgen.1006734.s010.rtf]

S3 Table: Description of plasmids used for U. maydis strain generation
Plasmid	pUMa	Resistance cassette	Short description	
pVps60D-HygR	pUMa1698	HygR (pStorI-1hs)	Plasmid used for vps60 deletion. The hygromycin-resistance cassete is flanked by 0,8 kb upstream and  by 1 kb downstream of the region surrounding vps60. The flanks were amplified by PCR (UF: oRL650 & oRL651; DF: oRL652 & RL653) on genomic 521 DNA, while the resistance cassette was derived from pStorI-1hs. All components were cloned into pDest (Terfrüchte et al., 2014) 	
pDid2D-HygR 	pUMa1699	HygR (pStorI-1hs)	Plasmid used for did2 deletion. The hygromycin-resistance cassete is flanked by 1 kb upstream and by 0,8 kb downstream of the region surrounding did2. The flanks were amplified by PCR (UF: oRL658 & oRL659; DF: oRL660 & RL661) on genomic 521 DNA, while the resistance cassette was derived from pStorI-1hs. All components were cloned into pDest (Terfrüchte et al., 2014) 	
pDid2G-NatR	pUMa1700	NatR (SfiI-insert of pMF5-1n)	Plasmid used to generate Did2-eGfp fusion. A eGfp-cassette containing eGfp, Tnos terminator and nourseothricin-resistance cassette is flanked by a 1.2 kb upstream and a 1 kb downstream flank. The upstream flank encompasses the entire did2 ORF and the region upstream of did2. The downstream flank contains the region directly downstream of did2. Both flanks were amplified by PCR on genomic UM521 DNA (UF: oRL 694/oRL695; DF: oRL696/oRL661).	
pRab5a-paG3-CbxR 	pUMa1477	CbxR (for integration at ips locus)	Plasmid used to ectopically express triple photoactivateable Gfp (paG3)-Rab5a. The 909 bp rab5a-ORF is fused C-terminally to pa-Gfp3. The construct is under control of the strong constitutive Potef-promotor and is flanked by two Tnos-terminators. (plasmid generated by S. Baumann)	
pPhoxG-CbxR 	pUMa2931	CbxR (for integration at ips locus)	Plasmid used to ectopically express Phox-Gfp. The  432 bp long Phox-domain was derived from Yup1 (aa 1-148) and amplified by PCR (oMF807 & oMB282) on a plasmid ectopically expressing Yup1-mCherry-myc. Phox-Gfp is under control of the constitutive Potef-promotor and flanked by the Tnos-terminator.	
pVps27G-NatR 	pUMa1714	HygR (SfiI-insert of pMF5-1h)	Plasmid used to generate Vps27-eGfp fusions. A eGfp-cassette containing eGfp, terminator Tnos and a hygromycin-resistance cassette is flanked by an upstream and downstream flank. The 1.1 kb upstream flank is comprised of a vps27 fragment, missing its 5´end. The 0.9 kb long downstream flank contains the genomic region immeadiately downstream of vps27. The corresponding regions were amplified by PCR (UF: oRL689 & oRL690; DF: oRL642 & oRL643) on genomic 521 DNA.	
pDid2D-G418R 	pUMa2452	G418R (SfiI-insert of pMF1-g)	Plasmid used for did2 deletion. The hygromycin-resistance cassette of pDid2D-HygR was exchanged with a geneticin-resistance cassette.  	
pVps4G-NatR 	pUMa1871	NatR (SfiI-insert of pMF5-1n)	Plasmid to generate Vps4-eGfp fusions. A eGfp-cassette containing eGfp, terminator Tnos and a nourseothricin-resistance cassette is flanked by an upstream and downstream flank. The 1.4 kb upstream flank is comprised of a 3´fragment of the vps4 ORF. The 0.6 kb long downstream flank contains the genomic region immeadiately downstream of vps4. The corresponding regions were amplified by PCR (UF: oDD703 & oDD704; DF: oDD705 & oDD706) on genomic 521 DNA.	
pDyn2G3-HygR 	pUMa965	HygR	Plasmid used to generate triple Gfp-Dyn2 fusion. Published in Lenz et al., 2006.	
pPep4D: Rab7G-NatR 	pUMa2666	NatR (SfiI-insert of pMF5-1n)	Plasmid used to ectopically express Gfp-Rab7 at the pep4-locus. Plasmid is based upon pPep4D-HygR (pUMa1646; Sarkari et al., 2014). The 0,7 kb long rab7-ORF containing Exons 1 and 2, is fused C-terminally to eGfp under control of the strong constitutive Po2tef-promotor. The construct contains a nourseothricin-resistance cassette and is flanked upstream by 0,8 kb and downstream by 0,7 kb of the immediate genomic region flanking pep4.	
pPrc1C-CbxR 	pUMa2135	CbxR (for integration at ips locus)	Plasmid used to ectopically express Prc1-mCherry ectopically at the ips locus.  mCherry is fused C-terminally to the 1,8 kb long prc1 ORF. The construct is flanked by the strong constitutive Potef-promotor and the Tnos transcriptional terminator.	
pCps1C-CbxR	 pUMa2743	CbxR (for integration at ips locus)	Plasmid used to ectopically express mCherry-Cps1 ectopically at the ips locus. The 1,8 kb long cps1-ORF  is fused C-terminally to mCherry and flanked by the constitutive Ptef-promotor and two copies of the Tnos transcriptional terminator. The cps1-ORF was amplified by PCR on genomic 521 DNA using oligonucleotides oDD725 and oDD726.	
pDid2-CbxR	 pUMa2710	CbxR (for integration at ips locus)	Plasmid used to ectopically express Did2 from the ips locus. The 0,6 kb long did2 ORF is flanked by the constitutive Ptef-promotor and the transcriptional terminator Tnos. The did2-ORF was amplifed by PCR on genomic 521 DNA using oligonucleotides oDD651 and oDD652.	
