# Supplementary material for: The ESCRT regulator Did2 maintains the balance between long-distance endosomal transport and endocytic trafficking
Source: PLoS Genet. 2017 Apr 19;13(4):e1006734. doi: 10.1371/journal.pgen.1006734 (PMC5415202; doi:10.1371/journal.pgen.1006734)
Supplement: S4 Table — (RTF) [file pgen.1006734.s011.rtf]

S4 Table: DNA oligonucleotides used in this study
Oligonucleotide	Sequence (5´- 3´)	Gene	
oRL650	GGTCTCGCCTGCAATATTGAAATCGAGCCGATTGGTC	vps60	
oRL651	GGTCTCCAGGCCGGTGAAAGAGTACGAGTAGATG	vps60	
oRL652	GGTCTCCGGCCGCGTCTTCTATTCAGATCCGC	vps60	
oRL653	GGTCTCGCTGCAATATTGCAGACGGTTCAACCGCG	vps60	
oRL658	GGTCTCGCCTGCAATATTTCTTGCACTCCCACCGTCG	did2	
oRL659	GGTCTCCAGGCCGATGCAGATACAGCCTTGCTG	did2	
oRL660	GGTCTCCGGCCAAGTCTCGACATGATCAAATTTTCG	did2	
oRL661	GGTCTCGCTGCAATATTAGCGCAGGAGTACGATCTCC	did2	
oRL694	AATATTCTGATCGTGTTGGGCAG	did2	
oRL695	GGCCGCGTTGGCCGCCGTGGCAGGTCGGAGTGCGC	did2	
oRL696	GGCCTGAGTGGCCAAGTCTCGACATGATCAAATTTTC	did2	
oMF807	TCCAATAAAGGGCGCTGTCTCGGC	Potef	
oMB282	CGGCCATGGCCCATTCGATAAACTGCTTGAACGC	yup1	
oRL689	GGTCTCGCCTGCAATATTCGGGTACTCCTGCTCCGTG	vps27	
oRL690	GGTCTCCTGGCCCGAAACTCGATCAAAGGCGATTCC	vps27	
oRL642	GGTCTCCGGCCAAAGTCGCCCATGTGATCTTGGC	vps27	
oRL643	GGTCTCGCTGCAATATTTTTCGAGAAGAAAGCCACAACGC	vps27	
oDD703	GGTCTCGCCTGCAATATTCGGATTTCTTGAATAAGG	vps4	
oDD704	GGTCTCTTGGCACCCGCCTCGTTGGTAAAC	vps4	
oDD705	GGTCTCAGGCCGCACTCAGTGTCTTCGTCTG	vps4	
oDD706	GGTCTCGCTGCAATATTCAGGGACGGGCAGCAC	vps4	
oDD725	ACGCGGCATATGGGTCGAATCGAAGAG	cps1	
oDD726	CAATGGCGCGCCCTAGAAATCTGTCTGCGACC	cps1	
oDD651	GCCTGCGAATTCATGTCGGGACTGGAAAAG	did2	
oDD652	TAATTCGGCGCGCCTTACGTGGCAGGTCGGAGTG	did2	
